# Theoretical study of the properties of X-ray diffraction moiré fringes. III. Theoretical simulation of previous experimental moiré images

**DOI:** 10.1107/S205327332000532X

**Published:** 2020-06-30

**Authors:** Jun-ichi Yoshimura

**Affiliations:** a Sakai 5-13-2-A322, Musashino-shi, Tokyo 180-0022, Japan

**Keywords:** X-ray moiré fringes, strained crystals, low-contrast band pattern, peculiar experimental fringe profiles

## Abstract

As a practical confirmation of a recently published X-ray moiré-fringe theory [Yoshimura (2015 ▸). *Acta Cryst.* A**71**, 368–381], computer simulations using this theory were conducted for previous experimental moiré images of a strained bicrystal specimen [Yoshimura (1996 ▸). *Acta Cryst.* A**52**, 312–325]. Simulated moiré images with a good or fairly good likeness are presented as a result of this simulation, in which the characteristic fringe-and-band and local strain patterns in the experimental images are reproduced well. Experimental moiré images taken when the inclination of the lattice planes was forcedly increased in one of the component crystals of the bicrystal specimen were also fairly well simulated in this computation, and their fringe patterns of inclined fringes are shown to be in accordance with the prediction by the theory. This moiré-fringe theory is thus considered to be widely applicable to the study of moiré images. Furthermore, the successful simulation of the previous experimental moiré images means that a satisfactory theoretical explanation was given for the experimental images, with respect to their characteristic global features. However, this study by the theoretical simulation shows explicitly that some significant peculiarities in the fringe profiles of the experimental images still remain unexplained by this moiré-fringe theory.

## Abstract 2

Using a recently developed moiré-fringe theory of X-ray diffraction, computer simulations of previous experimental moiré images are presented, for an experimental verification of the moiré-fringe theory and for a theoretical explanation of the peculiar experimental moiré images.

## 1. Introduction   

In Part I of this series of theoretical studies of X-ray moiré fringes (Yoshimura, 2015 ▸), we described the basics of the moiré-fringe theory and presented some examples of computed plane-wave moiré images that are considered to represent the basic characteristics of moiré images. In Part II of this series (Yoshimura, 2019*a* ▸), moiré images obtained through computations under more practical conditions of an incident beam with wider angular spreads were described, and it was shown how they change with such factors as the thicknesses of the specimen bicrystal, the width of an interspacing gap in the bicrystal, the angular width of the incident wave, the curvatures in the bicrystal *etc*. In this paper, *i.e*. Part III of the same series, theoretical simulations on the previous experimental moiré images (Yoshimura, 1993 ▸, 1996*a* ▸,*b* ▸, 1997*a* ▸,*c* ▸) are presented as a further application of this moiré-fringe theory. The experimental moiré images were taken under a quasi-plane-wave condition using an incident beam with a small angulular spread, and with a strained bicrystal used as the specimen. Comparison of simulated moiré images with the experimental images will serve as a check of the correctness of the moiré-fringe theory. On the other hand, this simulation work is an attempt to provide a full theoretical description of the experimental moiré images mentioned above, which have long been unexplained. This theoretical work will help to advance the discussion on the previous moiré-image experiment.

Herein, Part I, including the attached addenda-and-errata paper (Yoshimura, 2019*b* ▸), and Part II are referred to as Papers I and II, respectively. The equations and figures in Papers I and II are referred to as equation (I-*i*) and equation (II-*i*), and Fig. I-*i* and Fig. II-*i*, with *i* indicating the number of the equation or figure.

## 2. Description of this computer simulation work   

### 2.1. Experiment to produce and record moiré images   

#### 2.1.1. Experiment setup   

**Figure 1:**
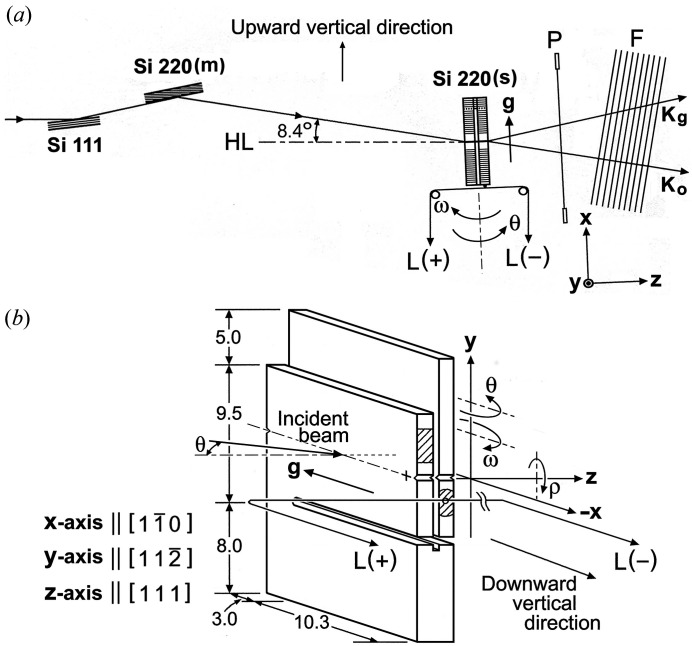
(*a*) General view of the experiment setup. Si 220 (*s*), specimen; 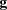, diffraction vector; **K**_*o*_ and **K**_*g*_, wavevectors of transmitted and of diffracted waves, respectively; the incident beam is σ-polarized (horizontally polarized). *P*, thin platinum wire stretched in a rigid frame; *F*, X-ray films, set perpendicularly to the **K**_*o*_ beam; 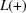 and 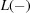, pulling weight for causing a forced rotation of the rear component crystal. The dashed line *HL* indicates the horizontal direction. The *y* axis is directed to a horizontal direction, the *x* axis is parallel to the diffraction vector 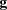, and the *z* axis is perpendicular to the crystal surfaces. The 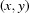 coordinate system is the same in the incidence surface of the front component crystal and in the exit surface of the rear component crystal; its origin is placed at the cross-marked position near the 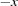-side edges of the component crystals, as shown in (*b*); the origin of the *z* coordinate is placed on the exit surface of the rear crystal. (*b*) Detailed drawing of the specimen bicrystal. Dimensions are given in mm. Circular curves 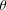 and 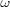 about the *y* axis illustrate, respectively, the diffraction angle (incident glancing angle to the diffracting lattice plane) and the 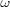 rotation [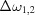, 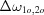; see equations (4*a*)[Disp-formula fd4a] and (4*b*)[Disp-formula fd4b] in the text] of the component crystals; circular curve 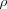 about the *z* axis illustrates the 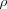 rotation (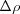, 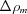 *etc*.; see Sections 2.1.3, 2.2.1 *etc*.) of the component crystals. In every circular curve the arrow indicates the positive direction of the rotation.

For an introduction to the moiré images which are the subjects of the simulation work, the experiment and the specimen crystal with which the moiré images were taken are outlined below, though they were described in the previous papers (*e.g*. Yoshimura, 1996*a* ▸). The experiment setup and details of the specimen crystal are shown in Fig. 1 ▸. The experiment was conducted using synchrotron radiation at Station BL-15C (the station name at the time) at the Photon Factory, KEK, Japan in 1989. Synchrotron X-rays (σ-polarized) which were monochromated and collimated by the Si 111 and Si 220 (*m*) monochromators struck the specimen crystal Si 220 (*s*). The wavelength of the incident beam was centred at λ_o_ = 0.072 nm, and is considered to have a small spread of 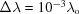 as a result of the monochromatization by the successive diffraction by Si 111 and Si 220 (*m*) [see Fig. 11 in Yoshimura (1996*a* ▸)]. The corresponding Bragg angle 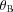 was 10.81°, and the angular width of the incident beam was 0.34′′, which was narrowed by the highly asymmetric diffraction at the monochromator Si 220 (*m*). For the diffraction at the specimen crystal, the 220 reflection was used in symmetric Laue geometry, the specimen being set in a parallel setting with the upstream Si 220 (*m*) monochromator. The beam was incident on the crystal surface at an angle of 8.4° from the horizontal [see Fig. 1 ▸(*a*)], and the specimen crystal was accordingly inclined by 2.4° from the vertical. Moiré images were taken at the peak position of the rocking curves for the diffracted beam, at a distance of 54–64 mm from the specimen. To record the diffracted images, single-coated X-ray films were specially prepared from conventional-type high-resolution films (Fuji type No. 50, with an undeveloped grain size of 0.3 µm). The exposure time was 25–35 s. The moiré images were simultaneously recorded onto eight to 12 films. Between the specimen and films, very thin Pt wires were stretched. The purpose of the simultaneous recording and the wire stretch was described in the previous papers (*e.g.* Yoshimura, 1996*a* ▸).

#### 2.1.2. Specimen crystal   

The specimen crystal was a monolithic bicrystal, the details of which are sketched out in Fig. 1 ▸(*b*). It was composed of a bicrystal part having an interspacing gap above the lateral cut (along the *x* axis) at a height of 8 mm from the bottom, and a single-plate part below the lateral cut. The reciprocal-lattice-vector difference 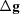 to produce the moiré fringes was mainly introduced through a minute relative rotation about the *z* axis between the two component crystals of the bicrystal. The crystal surface was parallel to the (111) plane, with the diffracting lattice plane 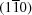 perpendicular to it (symmetric Laue geometry). The thicknesses of the front (incident-beam side) and rear (outgoing-beam side) crystals, and of the gap layer, were re-measured using a dial gauge to determine their exact values, after splitting the bicrystal into two single-plate crystals during this simulation work. (The reported thicknesses in the previous papers were estimated from the X-ray absorption rate.) The orientations of the interspacing gap surfaces were found to be slightly rotated by 0.37° (about the *y* axis) from the exact (111) plane, based on an X-ray orientation measurement. According to the definition given in Fig. I-3 in Paper I, this tilt angle is represented as α = −0.37° = −0.00646 rad. The thickness of the front crystal was 1605 µm, and that of the rear crystal 1517 µm. These values slightly increase or decrease in the *x* direction, reaching a total variation of 40–60 µm within the entire specimen width along the *x* axis. The thicknesses also varied in the *y* direction by 10–20 µm. The variation in the crystal thicknesses in the *x* direction was consistent with the misorientation of the ap space above (α = −0.37°). By subtracting the two crystal thicknesses above from the total bicrystal thickness 3365 ± 5 µm, the width of the gap space was estimated to be 243 µm.

**Figure 2:**
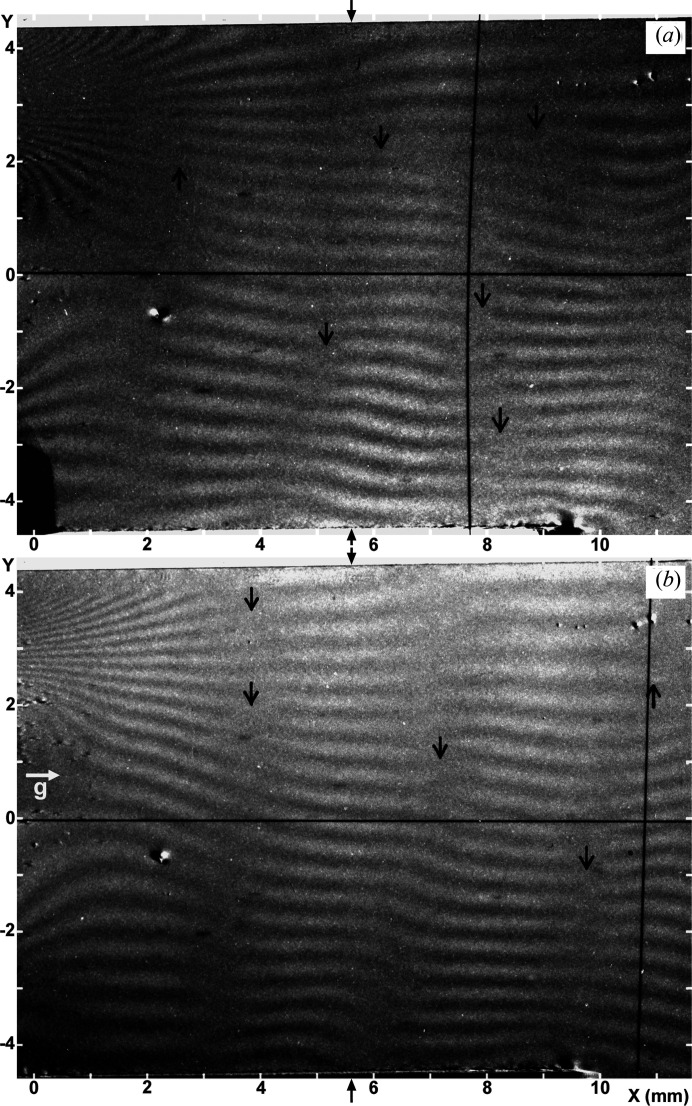
Experimental moiré images (T set No. 5). Si 220 reflection, λ = 0.072 nm. Taken with no forced rotation to the specimen, 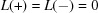. (*a*) *O* image, (*b*) *G* image. **g** is the diffraction vector. The 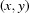 coordinates in the presented images are the same as those on the incidence and exit surfaces of the specimen crystal as described in the caption of Fig. 1 ▸. The lateral width (in the *x* direction) of the experimental *O* image is extended by 1.06 times that of the original as-recorded images by computer processing, so as to agree with that of the *G* image. Vertical arrows drawn in the images note the occurrence of pseudo moiré dislocations (see Section 3.1.4). Smaller vertical arrows drawn to the *x*-coordinate axes outside the images indicate the position of *x* = 5.6 mm at which the intensity profiles in Fig. 4 ▸ were measured. The long horizontal and vertical black lines are the shadows of the thin platinum lines stretched between the specimen and films, as mentioned in Section 2.1.1. [Similar black lines seen in Figs. 6 ▸(*a*), 7 ▸(*a*) and 9 ▸ are all of the same origin as above.] The black region in the lower-left corner in the *O* image is the shadow of a pillar for holding the wire of the pulling weight [see Fig. 1 ▸(*a*)]. For other details, see text.

In addition, near the midpoint within the entire *x* dimension of the specimen on the inner surface of the front crystal, an abrupt step-like thickness change of about 20 µm was found, which is considered to have been made while sawing the specimen for the gap space. Regarding this change in thickness, a noticeable feature in the moiré images will be shown later in Fig. 2 ▸(*b*). Although such small thickness variations were unexpectedly found, the computer simulation was conducted with the model that the two component crystals and the gap space are of a uniform thickness, ignoring their small variations. The small misorientation above α = −0.37° of the inner gap surfaces was considered only in the calculation of the moiré-interference phase in equation (8). As with the outer surfaces, the inner gap surfaces of the bicrystal were polished and etched to remove the stresses and strains.

#### 2.1.3. Relative rotations between the component crystals and other strains in the specimen crystal   

(i) The two component crystals were minutely rotated about the *z* axis by gravity, accompanied by an elastic bend at their supporting sites, and a fringe pattern of rotation moiré was expected to be produced owing to the difference between their rotation angles. The moiré experiment was planned to produce moiré fringes with a spacing of 0.4–0.6 mm. The adjustment to the target fringe spacing was attained by attaching a balancer weight of 0.236 gf (gram-force) to an upper position on the front crystal [hatched portion in Fig. 1 ▸(*b*)] by gluing (using Araldite). Although it had been implicitly thought that the rotation (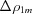) of the front crystal did not exceed that (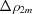) of the rear crystal in this adjustment, it was found in the present study that the weight of the front crystal with the balancing weight slightly exceeded the weight of the rear crystal. This was known from the measurement of the weight of the two component crystals after splitting the bicrystal. According to this reassessment, the sense of the relative rotation between the two component crystals about the *z* axis, which provides the main component of the reciprocal-lattice-vector difference for moiré fringes, was presumed to be 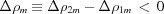 (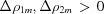). The correctness of this presumption is reconfirmed through the description of the moiré images in Section 3.1.3.

(ii) In addition to the 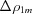 and 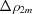 rotations about the *z* axis in the respective component crystals, the rear crystal was forcedly rotated about the *y* axis by a minute angle, when recording some of the moiré images [Figs. 6(*a*) and 7(*a*)]. The forced rotation was made by gluing a thin wire onto the edge of the rear crystal, the two opposite ends of which were connected to a pulling weight [*L*(+) or *L*(−) in Fig. 1 ▸]. The purpose of the forced rotation of the rear crystal is described in Section 4.1.1.

(iii) The fixing of the balancing weight and the fine wire to the crystal edges, as described above, induced strain (lattice contraction) in the specimen, causing a local modulation of the moiré-fringe pattern, which was an unwelcome result. Although it was once considered a difficult problem to estimate precisely the induced strains for simulation computations, the problem was mostly solved through the elasticity theory (see Appendix *A* [App appa]). Hereafter, these local strains from the left edges (the −*x* side) of the component crystals are referred to as LEC local strains.

(iv) In addition to the strains described above, a weak curvature strain (0.01–0.07′′ mm^−1^) about the *y* axis had been suggested to occur in the specimen crystal, from the presence of low-contrast band images like equal-inclination fringes in the experimental images. This curvature strain was the most dominant strain in the specimen, as will be shown later.

(v) From the observation that the moiré-fringe spacing increases slightly in proceeding to the top in the images [see Figs. 2 ▸(*a*) and 2 ▸(*b*)], it had been noticed that a very weak bending about the *z* axis was induced by gravity along the *y* direction in the component crystals, accompanying their 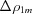 and 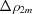 rotations. According to elasticity theory on the bending-of-bar problem (*e.g*. Takeuchi, 1969 ▸), the longer crystal (the rear crystal in this case) bends more than the shorter one (front crystal) along their length (in the *y* direction), since the length of the bar works more effectively than its weight. Such a difference in the bend deformation between the component crystals relaxes the effect of the relative 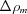 rotation so that the fringe spacing is increased.

(vi) Nicks [see Fig. 1 ▸(*b*)] were purposely made on the right and left edges of each component crystal as positional marks. They unhelpfully affected the strain distribution around them, and thereby disturbed the local fringe pattern. This strain disturbance was not taken into account in this simulation, because it was limited to a very small area and was difficult to deal with theoretically.

(vii) As mentioned in Section 2.1.1, the component crystals were inclined from the vertical line by 2.4° in the counter-clockwise direction during the experiment. Accordingly, a torsional rotation by gravity is considered to have been induced in the crystals, although no clear evidence for this rotation was found in the present experimental images. When the inclination from the vertical line is increased, this effect comes to be clearly observed.

### 2.2. Method for computing simulated images   

#### 2.2.1. Equations for simulation computations   

Computations of the simulated moiré images were conducted as an angular integration of plane-wave image intensity, in the same way as applied in the preceding computations described in Paper II [see equation (II-1)]. The intensities of *O* (transmitted-wave) and *G* (diffracted-wave) images were computed, respectively, using the following equations: 
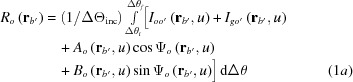


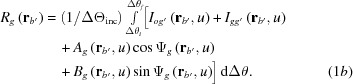
Here, the argument 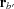 denotes a vector referring to a position on the exit surface of the rear crystal; 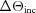 denotes the angular width of integration 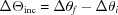, and in the present case 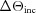 = 0.34′′ = 1.648 × 10^−6^ rad; 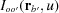, 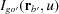, 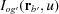 and 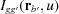 represent a partial image intensity unrelated to the moiré interference; 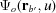 and 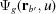 denote the phases of a moiré-fringe interference. The full expressions of 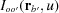, 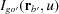, 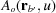, 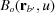 and 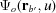 in equation (1*a*)[Disp-formula fd1a] are given in equations (I-51*a*)–(I-53) in the supporting information to Paper I (Yoshimura, 2019*b* ▸). The full expressions of 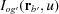, 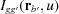, 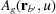, 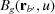 and 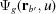 in equation (1*b*)[Disp-formula fd1b] are given in equations (I-22*a*)–(I-23*b*) and (I-34) in Paper I. The variable of integration 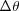 denotes the deviation angle from the exact Bragg position when the X-ray wave is incident on the front crystal of the bicrystal (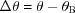, 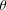 being the incidence glancing angle to the diffracting lattice plane) (see Appendix *B* [App appb] for list of symbols).

The deviation parameter of diffraction *u*, which corresponds to the deviation angle 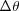 above, is given as follows, in agreement with equation (I-45) in Paper I: 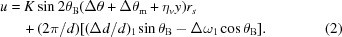
Here, we assume symmetric Laue geometry in agreement with the experimental condition; *K* is the wavenumber; 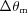 indicates the middle position in the integration width 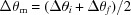, which hereafter is called the mid-deviation angle (at 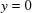); here and hereafter, the representation of position variable 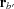 is replaced with 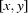 (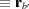), *x* and *y* being given in units of mm; 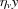 represents a variation in the effective deviation angle owing to the vertical divergence of the beam, 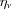 being the rate of the deviation-angle variation (= 0.028′′ mm^−1^) (see in detail in Section 2.2.2.). Through the addition of this angular variation 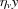, 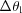 and 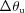 in the integration of equations (1*a*)[Disp-formula fd1a] and (1*b*)[Disp-formula fd1b] vary to 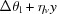 and 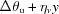, respectively, and the mid-deviation angle varies to 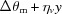; 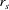 means the conversion factor 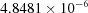 from arcseconds to radian. The symbol *d* means the lattice spacing; 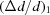 and 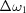 denote a local variation in the lattice spacing and a local inclination of the diffracting lattice plane (see Fig. 1 ▸) in the front crystal, respectively. Herein, the subscript indices 1 and 2 refer to the front and rear crystals, respectively.

The deviation parameter with respect to diffraction in the rear crystal is given as follows, succeeding to *u* in equation (2), and in agreement with equations (I-16*a*) and (I-16*b*): 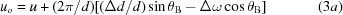


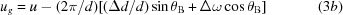
where 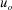 and 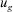 are the deviation parameters with respect to the diffraction of waves propagated in the transmitted- and diffracted-wave directions, respectively, after emerging from the front crystal. Here, 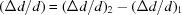 and 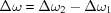, 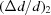 and 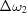 being a local variation in the lattice spacing and a local inclination of the diffracting plane in the rear crystal, respectively.

The local inclinations of the diffracting lattice plane 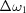 and 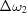 are given as follows: 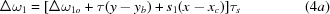


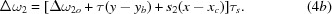
Here, 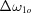 and 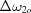 denote invariable parts of the lattice-plane inclinations; 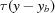 represents the torsional rotation about the *y* axis presumed from the 2.4° tilt of the component crystals, as mentioned in Section 2.1.3, and the magnitude of 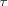 was assumed to be 0.003′′ mm^−1^; 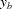 means the *y* coordinate of the starting position of the torsional rotation, and was assumed to be 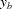 = −4.7 mm; 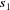 and 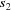 denote the strength of the curvatures of the front and rear crystals, respectively; 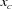 denotes the *x* coordinate where the bend of the diffracting plane due to curvatures 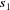 and 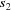 becomes zero, which was set to be 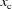 = 9.5 mm after trying several likely values during the simulation computations. The variation in the lattice spacing is only that from the LEC local strains, and was given by 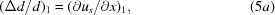


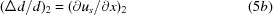
from the solutions in the elasticity calculation (Appendix *A* [App appa]). Here 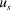 denotes the displacement in the *x* direction, and the expression of 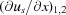 is given in equation (10*a*)[Disp-formula fd10a].

The relative rotation 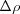 about the *z* axis between the component crystals, which is responsible for the rotation-moiré pattern, was given as follows: 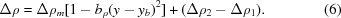
Here, 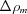 represents the main relative 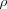 rotation between the component crystals of the bicrystal, as mentioned in Section 2.1.3. Its value was taken to be 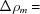 −*d*/0.44 mm (= −0.436 × 10^−6^ rad = −0.090′′) throughout all simulation computations in this paper, as determined from a comparison of many simulated images with the corresponding experimental images; this value corresponds to the fringe spacing of Λ = 0.44 mm. The correction factor 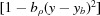 is related to the weak bending in the component crystals, as mentioned in item (v) in Section 2.1.3; 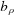 is an adjustment constant of the bend strain (see further Section 3.1.4). The added rotations, 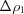 and 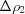 above, are the rotation of the lattice planes caused by the LEC local strains, and are given by 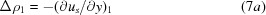


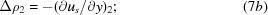
here, 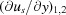 are the solution of the elasticity calculation, given in equation (10*b*)[Disp-formula fd10b].

The moiré-interference phases 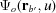 and 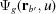 in equations (1*a*)[Disp-formula fd1a] and (1*b*)[Disp-formula fd1b] were given by 
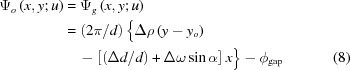
[see equations (I-34), (I-53) and (II-8)]. Here, the introduction of an origin 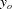 in the term 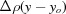 is explained in the simulation of the fringe profiles in Fig. 5; the contribution of the term 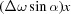 is very small for the images in Figs. 2 ▸ and 3 ▸ with 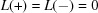 (Section 3.1), but becomes significant in the discussion of Figs. 6 and 7 with 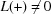 or 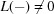 (Section 4.1). The term 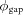 denotes the gap phase.

Based on the theoretical preparations as above, the numerical integration of 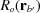 and 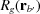 in equations (1*a*)[Disp-formula fd1a] and (1*b*)[Disp-formula fd1b] was made with an angular step of 0.01′′, using *Visual Basic .NET* Version 2003 software. The computed intensity of the moiré images was multiplied by the correction factor 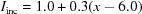, corresponding to a non-uniformity in the *x* direction in the incident-intensity distribution from the Si 111 → Si 220 (*m*) monochromator system (Fig. 1 ▸). The non-uniformity was determined from a comparison of the intensity distributions in the simulated and experimental images. The values of the basic constants of X-ray diffraction, namely, 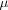, 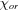, 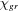, 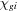 were calculated for 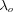 = 0.0720 nm and other tentatively used wavelengths, following the *International Tables for Crystallography* Vol. C (Wilson, 1995 ▸). (Here, 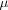 is the linear absorption coefficient, and 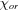, 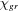 and 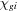 are a real or imaginary part of the Fourier components of dielectric susceptibility.)

#### 2.2.2. Effect of vertical divergence of the beam   

An unexpectedly large amount of time had to be spent to accomplish this simulation work. A major cause was the difficult problem imposed by the effect of a vertical divergence of the beam. Here, a vertical divergence effect means that the effective deviation angle varies in the vertical direction in single-crystal X-ray diffraction in multiple-crystal arrangements (Jäger, 1965 ▸, 1966 ▸; Yoshimura, 1984 ▸). When a multiple-crystal arrangement involves a non-parallel setting as its element, the variation of the effective deviation angle becomes significant. In the experiment under study, the arrangement Si 111 → Si 220 (*m*) was of such a non-parallel setting (see Fig. 1 ▸). The word ‘vertical’ refers herein to the *y* direction. Factors affecting the magnitude of the vertical divergence effect in synchrotron-beam diffraction are now considered to be the source-to-specimen distance and the magnitude of directivity or angular divergence of the beam from the source. However, when the experiment was conducted, the author had no recognition of the latter factor. The ratio of the specimen size to the source-to-specimen distance 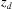 was sufficiently small (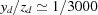, with 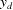 and 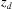 being 10 mm and 30 m, respectively), and therefore the vertical divergence effect was considered to be negligible. However, in the inspection of the images obtained after the experiment, an unexpected common feature was noticed in their intensity distributions: the intensity in the *G* images increases towards the +*y* direction, and the intensity in the *O* images decreases towards the same direction [see Figs. 2 ▸(*a*), 2 ▸(*b*) and Figs. 6(*a*) and (7*a*)], although normally the image intensity should be almost constant in the *y* direction, under a constant mid-deviation angle 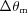. This intensity variation should necessarily have been taken to mean that the effective deviation angle is varied so as to increase towards the +*y* direction.

In subsequent synchrotron experiments, the vertical divergence effect was more carefully and clearly ascertained as a definite experimental fact, although the theoretical reason for it was still unknown. In 2008, the author knew the importance of one more factor, the directivity of the beam (*e.g.* Ohhashi & Hirano, 2008 ▸), and reached a qualitative understanding on the operation of an effective vertical divergence effect in synchrotron-beam diffraction. As a written report, the operation of this effect in synchrotron experiments has been mentioned by Yoshimura & Hirano (2014 ▸). While the radiation of X-rays from a laboratory source is isotropic, a synchrotron beam has a high directivity (directional angular divergence of 10^−4^ rad). Owing to this high directivity, or the beam divergence in a very narrow angular width, the vertical divergence effect is considered to be severe in synchrotron-beam diffraction, in spite of the small value of the *y*_*d*_/*z*_*d*_ ratio. So far as the author knows, the much-needed exact theory dealing with the vertical divergence with synchrotron radiation has not yet been given anywhere. However, a practical estimation of the effect can be made expediently by comparing the diffracted intensity from a perfect crystal with a calculated rocking curve. Through such an estimation, the effective deviation angle is presumed to have varied by 0.3–0.4′′ over the entire *y* dimension (= 9.2 mm) of the moiré images, in this experiment.

Under the condition in which the deviation angle varies in the *y* direction, it was not easy to compute the moiré images that simulated well the experimental images in Figs. 2 ▸(*a*), 2 ▸(*b*), where characteristic low-contrast vertical bands run parallel from the bottom to the top in the images. After vain efforts, a way to solve this difficulty was found by trying computations at wavelengths other than the stated wavelength of 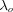 = 0.072 nm, and through computations with the range of view of the images extended imaginarily to broader dimensions (see Fig. 8). Through these trials, it came to be seen that good simulated images with long vertical bands appear in some limited area in the extended range of view, when appropriate values are assumed for such factors as 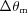, 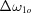 and 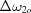. An appropriate combination of the values of the crystal thicknesses 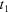 and 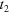, and the gap width 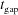 was also important. Although the variation in the deviation angle was initially estimated to be 0.3–0.4′′, as mentioned, the angular variation of 0.26′′ (= 0.028′′ mm^−1^ × 9.2 mm) was the limit of variation within which tolerably good simulated images can be obtained. Although trial simulations were conducted at different wavelengths, the final conclusive images were computed with the initial wavelength 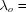 0.072 nm.

#### 2.2.3. Determination of the senses and strengths of curvatures in component crystals   

After solving the problem of the vertical divergence effect, we still had to continue a time-consuming computational study. A major problem then was to determine the senses and strengths of the curvatures 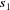 and 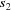 in the respective component crystals. As mentioned in item (iv) in Section 2.1.3, the occurrence of the curvatures 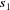 and 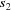 is suggested from the presence of low-contrast band patterns like equal-inclination fringes, which develop over the entire field of view of the experimental moiré images [see Figs. 2 ▸(*a*), 2 ▸(*b*) *etc*.]. The occurrence of such equal-inclination fringes in a moiré-fringe pattern, as a kind of *Pendellösung* interference fringe, has been described in detail in Section 3.2.4 in Paper II; speaking exactly, the gap phase 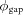 also takes part in the formation of the low-contrast bands in this case, along with the equal-inclination interference phase. Equal-inclination fringes are related to the local variation in the image intensity [see equation (II-13)], and therefore are related to the crystal curvatures 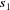 and 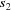, and with the lattice-plane inclinations 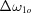 and 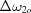, through 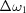 and 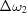 in equations (4*a*)[Disp-formula fd4a] and (4*b*)[Disp-formula fd4b]. The values of 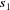 and 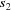, and of 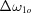 and 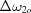 were adjusted and determined so that the positions and spacing of low-contrast bands agree well with those in the experimental images.

When the work of finding the solution values of 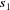 and 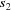 started, the only clue we had was an empirical law that, with the curvatures of 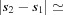 0.05′′ mm^−1^, three vertical bands are produced per ∼10 mm width in the *x* direction (in the case of the Si 220 reflection with a wavelength of about 0.071 nm, 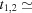 1.5 mm and 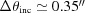); this law was derived from many simulation computations in Papers I and II. There was no clue about the signs of 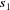, 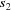 and (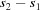). Therefore, the study had to account for all possibilities of the signs of the curvature values. Furthermore, the determination of the values of 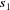 and 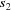 had to be made consistently through the simulations of all the images shown in Figs. 2 ▸(*a*), 2 ▸(*b*) [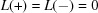], and Figs. 6(*a*) [*L*(+) = 0.2 gf] and 7(*a*) [*L*(−) = 0.2 gf]. To find the solution of this problem, trial-and-error computations were performed. After time-consuming computation work, we finally arrived at a convincing conclusion to the consistent values of 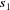 and 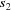. The study in an imaginarily extended range of view of images, as mentioned in Section 2.2.2, provided a good guidance to the solution. Added knowledge on the relation between the low-contrast bands and the values of 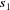 and 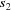 is shown in Fig. 8, as a result of the present study.

## 3. Results of computer simulations I   

### 3.1. Moiré images in the case of no forced rotation to the component crystals   

#### 3.1.1. General observations of the experimental images and solution of the corresponding simulated images   

Examples of the experimental moiré images are shown in Figs. 2 ▸(*a*) and 2 ▸(*b*). They were taken simultaneously under the same conditions in the experiment [with 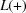 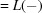 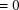]. They may be regarded as representative moiré images obtained in the experiment under study. The images are presented in such a way that they are viewed from the emerging-beam side. The image contrast is reproduced in such a way that white contrast indicates a higher intensity, which is opposite to the major convention.

The fringe patterns in the images in Figs. 2 ▸(*a*) and 2 ▸(*b*), roughly, are those of parallel moiré, but fringes somewhat slope upwards or downwards proceeding to the left edge, owing to the effect of the LEC local strain 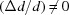. Strong contraction of the fringe spacing seen around 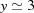 and 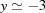 (mm) near the left edge of the images shows that a large 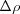 rotation is induced there in connection with strong contractions of the crystal lattice in the *y* direction (see Section 3.1.3 later). Diffuse, nearly vertical band images of weak contrast are considered to be equal-inclination interference fringes as mentioned in Sections 2.1.3 and 2.2.3. They are positioned at 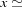 1.8, 4.3, 8 mm in the *O* image [Fig. 2 ▸(*a*)], and at 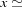 3, 5.8, 9.2 mm in the *G* image [Fig. 2 ▸(*b*)], as measured at the bottom of the images. Moiré fringes bend locally near the band images, which shows that the moiré fringes are strongly influenced there by the phase of the equal-inclination fringes; conversely this interaction shows that the band images are of equal-inclination fringes (see Section 3.2.4 in Paper II). In accordance with the explanation of a weak bending about the *z* axis along the *y* direction of the component crystals, in item (v) in Section 2.1.3, a small increase of the fringe spacing proceeding to the top in the images is readily seen. The short vertical arrows note the occurrence of pseudo moiré dislocations.

**Figure 3:**
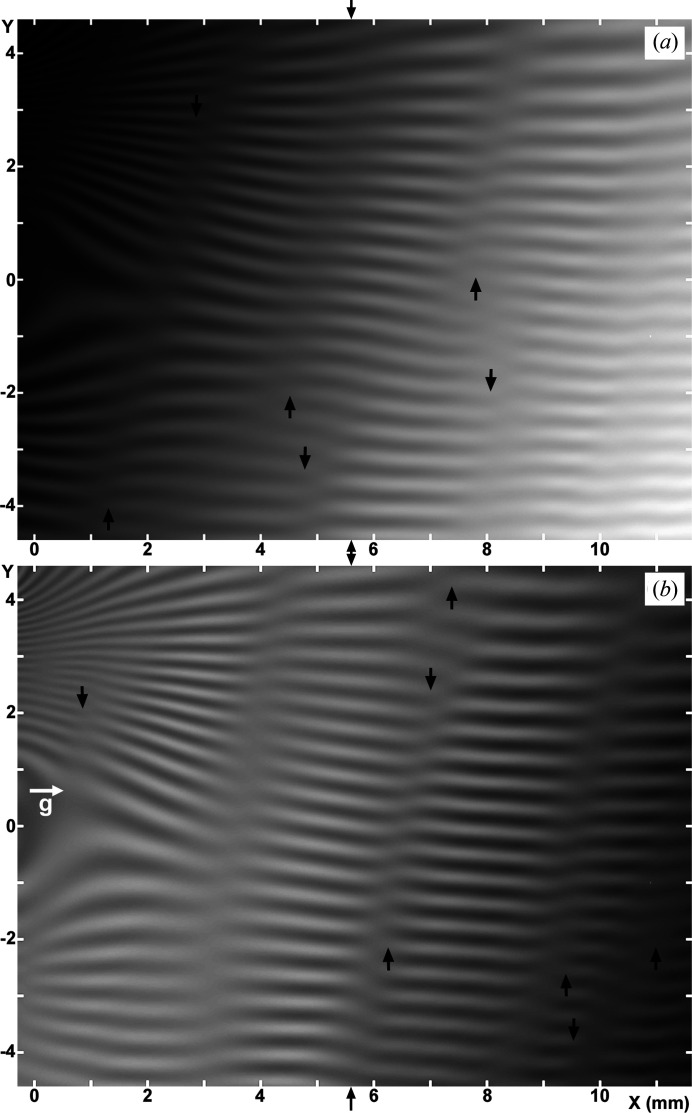
Computer-simulated moiré images corresponding to the experimental images in Fig. 2 ▸. (*a*) *O* image, (*b*) *G* image. The meanings of vertical arrows drawn in the images and smaller arrows outside the images are the same as those of the corresponding arrow groups in Fig. 2 ▸. See Section 3.1 and Table 1 ▸ for the parameter values of the computation and further explanation.

The best simulated images for the experimental images in Figs. 2 ▸(*a*) and 2 ▸(*b*), which were obtained as a conclusive result in this computational study, are shown in Figs. 3 ▸(*a*) and 3 ▸(*b*). The *O* [Fig. 3 ▸(*a*)] and *G* [Fig. 3 ▸(*b*)] images were computed under the same numerical conditions. The values of the parameters adopted in this computation of Figs. 3 ▸(*a*) and 3 ▸(*b*) are given in Table 1 ▸. The values of 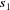, 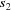 and 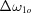, 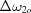 were determined as described in Section 2.2.3, with attention paid to the aspect of low-contrast band images. The allowable error limit to obtain tolerably good images was about ±0.002′′ mm^−1^ for 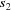 and ±0.001′′ mm^−1^ for 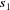. The value of the mid-deviation angle (at 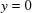) 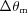 is involved in determining the *y* range of the field of view of the images, while also being used in determining the *x* position of the band images. After the values of 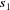, 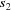 and 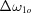, 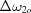 were approximately determined, details of the fringe-and-band pattern of the images were adjusted by changing incrementally the values of crystal thicknesses and the gap width 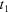, 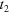 and 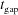. Through a change of 5 µm or less in 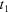 and/or 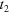, a significant change in the fringe-and-band pattern resulted. As a result of such fine adjustments, the best values of 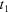, 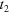 and 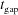 were determined conclusively to be 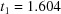, 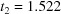, and 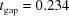 (in mm), although they slightly disagree with the values described in Section 2.1.2. The 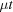 values then were 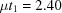 and 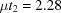. The number of fringes was adjusted so as to agree well with that in the experimental images (about 20 fringes, except the ones in the upper-left region in the image), by adjusting the value of 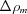; this value was eventually taken to be 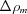 = −*d*/0.44 mm = −0.090′′, as stated in Section 2.2.1.

Based on the parameter values (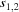, 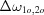) given in Table 1 ▸, the inclination (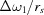) of the front crystal about the *y* axis is presumed to have varied from −0.1′′ (at 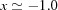) to +1.0′′ (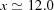), and that of the rear crystal (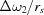) is presumed to have varied from −0.4′′ (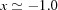) to +0.4′′ (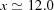). These variations with the *x* position in the (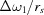) and (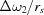) angles, and the curvature values of 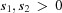 in Table 1 ▸, indicate that both the component crystals are curved concavely towards the 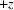 direction (see Fig. 1 ▸). Although the same sense of the two curvatures once seemed inexplicable, it is understandable if the curvatures were caused by the sawing process of the lateral cut in the specimen, along the *x* axis. With the 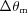 value in Table 1 ▸, the effective deviation angle 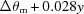 [the middle angle in the integration width in equations (1*a*)[Disp-formula fd1a] and (1*b*)[Disp-formula fd1b]] is presumed to have varied from −0.16′′ (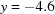) to +0.10′′ (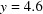).

#### 3.1.2. Comparison of the experimental and simulated moiré images. Patterns of low-contrast bands   

In the *O* image in Fig. 3 ▸(*a*), roughly vertical stripes or band images are observed at 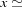 1, 5, 8 mm, as measured at the bottom edge of the image. In the *G* image in Fig. 3 ▸(*b*), they are seen at 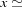 3, 5.8, 8.3, 9.8 mm. Although the positions of the band images do not exactly agree with those in the experimental images in Figs. 2 ▸(*a*) and 2 ▸(*b*), the fringe-and-band patterns in Figs. 3 ▸(*a*) and 3 ▸(*b*) may be considered to simulate well the fringe-and-band patterns in the respective experimental images, on the whole. The simulation of the band images in the *O* image, however, could not be very satisfactory, since the positions of simulated band images are displaced from the right positions, and a step-like refraction occurs in the upwards extension of one band. These unfavourable features in the simulated *O* image could be reduced to obtain a better simulation, if we shift the mid-deviation angle 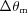 to the high-angle side. However, in this case, the simulation of the *G* image becomes worse in turn. Thus, the angular ranges for the best fit in the simulations of the *O* and *G* images did not match each other well. In this study, obtaining a good simulation for the *G* image was preferentially aimed for first.

As described in Section 3.2.4 in Paper II, and noted in the experimental images in Section 3.1.1 in this paper, moiré fringes in Figs. 3 ▸(*a*) and 3 ▸(*b*) also show a sharp bend in different degrees upon crossing the band image, and the fringe contrast drops down there. As mentioned already, the band images are of an analogous nature to the equal-inclination fringes. In accordance with the tentative nomenclature in Paper II, the band images in this paper are also called low-contrast bands (LC bands).

For a full appreciation of this simulation result, a comment should be made on the particularly large bend of moiré fringes seen around the LC band at 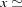 6 mm in the experimental *G* image in Fig. 2 ▸(*b*). A good simulation for this large fringe bend was difficult to attain in spite of the many trials [compare Fig. 3 ▸(*b*) with Fig. 2 ▸(*b*)]. Consequently, the large fringe bend is surmised to be connected with a special condition at the site in question. The abrupt thickness change of approximately 20 µm found at nearly the same position on the inner surface of the front crystal, as mentioned in Section 2.1.2, is presumed to be connected with this large fringe bend. A large and abrupt change in the *Pendellösung* interference phase, which would be caused by the large change in thickness, is surmised to have made the fringe bend so large. In the corresponding *O* image [Fig. 2 ▸(*a*)] and in other images shown later [Figs. 6(*a*) and 7(*a*)], no corresponding special image is seen in the area in question, suggesting that the large fringe bend was not due to strain.

#### 3.1.3. Comparison of the experimental and simulated moiré images. Influence of the LEC local strains   

The disturbance of the moiré patterns in the experimental images [Figs. 2 ▸(*a*), 2 ▸(*b*)], caused by the LEC local strains, may be assessed as being pretty well simulated in Figs. 3 ▸(*a*), 3 ▸(*b*), based on a general inspection. The expressions of the strain components used in the simulation computation are given in equations (10*a*)[Disp-formula fd10a]–(10*d*)[Disp-formula fd10d] in Appendix *A* [App appa]. Owing to a lack of space, the strain curves are not shown herein. The values of the strains were, for example, 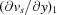 = −0.86 × 10^−6^ and 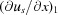 = 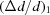 = 0.094 × 10^−6^ at *x* = −1.0, *y* = 3.4 (mm) in the front crystal (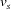 is the displacement in the *y* direction); and at *x* = 0.0, *y* = 3.4 (mm), 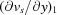 = −0.46 × 10^−6^, 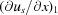 = −0.049 × 10^−6^ and 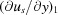 = 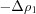 = −0.26 × 10^−6^. The curves (not shown) for the LEC local strains as a function of the *x* coordinate change considerably with the *y* position; as easily seen, in 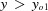 (= 2.4 mm), 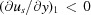, and 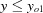, 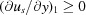 (for the meaning of 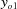, see Fig. 10 in Appendix *A* [App appa]). The local strains attenuate rapidly with the distance from the left edge of the crystal, but still have a magnitude nearly equal to or larger than 0.1 × 10^−6^ rad in the central region (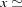 5 mm) of the specimen, and have a magnitude of 0.05 × 10^−6^ rad ≃ 0.01′′ near the right edge of the specimen. In the rear crystal the local strains were estimated for example as follows: 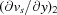 = −0.031 × 10^−6^, 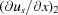 = 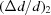 = −0.0089 × 10^−6^ and 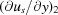 = 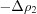 = 0.032 × 10^−6^ at *x* = 0.0, *y* = −3.0 (mm). The influence of the local strain in the rear crystal is limited to an area close to the left edge (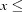 1 mm).

Table 2 ▸ shows an example [at *x* = 0.0, *y* = 3.4 (mm)] in which the value of 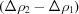 was determined from those of LEC local strains, and the total 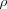 rotation, 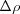, and the corresponding fringe spacing 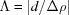 were obtained in accordance with it [the value of 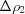 was 0.0079 × 10^−6^ in this evaluation of (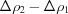)]. Furthermore, the results of the two cases, where 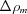 = −0.44 × 10^−6^ is assumed and where 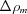 = +0.44 × 10^−6^ is assumed, are checked. In the former assumption the fringe spacing should be decreased to 0.28 mm, whereas in the latter assumption it should be increased to 1.03 mm. Since the actual fringe spacing decreases in the upper-left corner in the images, compared with the fringe spacing (≃0.44 mm) in other regions, the result (Λ = 0.28 mm) of the former assumption is affirmable. Thus, the correctness of the assumption of 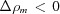, mentioned in Section 2.1.3, is definitely confirmed here.

However, this value 0.28 mm of fringe spacing still disagrees significantly with the actual fringe spacing 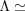 0.14 mm or 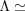 0.10 mm, which is observed in the same corner region in the simulated [Fig. 3 ▸(*b*)] or experimental [Fig. 2 ▸(*b*)] image. When we plot a curve of the interference phase 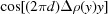 around *x* = 0, with 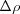 given as a function of position *y*, oscillations of almost the same spacing with 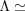 0.14 mm were observed near *y* = 3.4 mm. This result supports the correctness of the actual fringe spacing 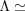 0.14 mm in the simulated images above. In this case the relative 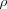 rotation by the LEC local strain, 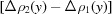, can be approximated by a linear function (in the region 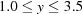), through the calculation using equation (10*b*)[Disp-formula fd10b] as follows: 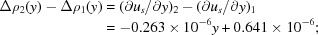
accordingly, the total relative 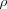 rotation in the interference phase is given as 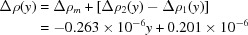
with 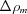 = −0.44 × 10^−6^; at *y* = 3.4, 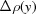 = −0.69 × 10^−6^ in accord with the value in Table 2 ▸. Thus, the interference phase in 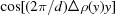 becomes a quadratic function of *y*, and the phase variation with position *y* is accelerated so as to produce a narrower fringe spacing than that by a simple estimation by the formula 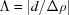. This property should be remembered when we deal with moiré fringes from a crystal having an inhomogeneous strain.

As to the experimental spacing 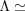 0.10 mm, almost the same fringe spacing was simulated when a curve of 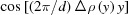 was plotted, with the term 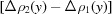 multiplied by 1.6 on trial; this result shows that the exact estimation of the LEC local strain would be 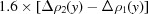, in the upper-left corner area. The presumed strong strain in this local area, which exceeds the strain value estimated from the ordinary linear elasticity theory, is surmised to be related to an effect of higher-order elasticity.

Although the fringe spacing differs significantly between the simulated and experimental images at 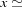 0.0 mm, the number of fringes over the entire *y* range in the simulated images in the region 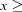 1.0 mm agrees well with that in the experimental images with a difference of less than one fringe.

#### 3.1.4. Comparison of the experimental and simulated moiré images. Indications of other strains   

Regarding the small increase in the fringe spacing towards the top in the images, which was noted in the observation of the experimental images in Section 3.1.1, the multiplication factor in the correction factor 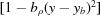 in the equation of relative 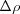 rotation in equation (6) was determined to be 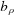 = 0.003 mm^−2^ for the conclusive simulated images in Figs. 3 ▸(*a*) and 3 ▸(*b*), by comparing trial simulated images with the experimental images in Figs. 2 ▸(*a*) and 2 ▸(*b*). An easily recognizable disagreement in the local fringe pattern around *y* = 0 on the left edge between the experimental and simulated images would be due to the neglect of the influence of small nicks at *y* = 0 in the simulation computation.

The short vertical arrows in Figs. 3 ▸(*a*) and 3 ▸(*b*) note the occurrence of pseudo moiré dislocations (Yoshimura, 1996*b* ▸; hereafter, PMD), similar to those in the experimental images in Figs. 2 ▸(*a*) and 2 ▸(*b*). As shown, many PMD images appear near or on the LC bands, and their positions are different between the *O* and *G* images. Such a characteristic of PMDs indicates that they are not directly connected to a disorder in a crystal lattice like real dislocations, but are connected with a condition of the *Pendellösung* interference phase which is influenced by macroscopic strain. They may be regarded as a common feature in moiré images of a strained crystal, when taken with a plane or quasi-plane incident wave. Disagreement in the positions of the PMDs between the experimental and simulation images strongly indicates the insufficiency of the present simulation of the experimental images. To make a more complete simulation of the occurrence of PMDs, a more accurate determination needs to be given of the strain distribution in the specimen.

### 3.2. Comparison of fringe profiles in the experimental and simulated moiré images   

#### 3.2.1. General observations   

**Figure 4:**
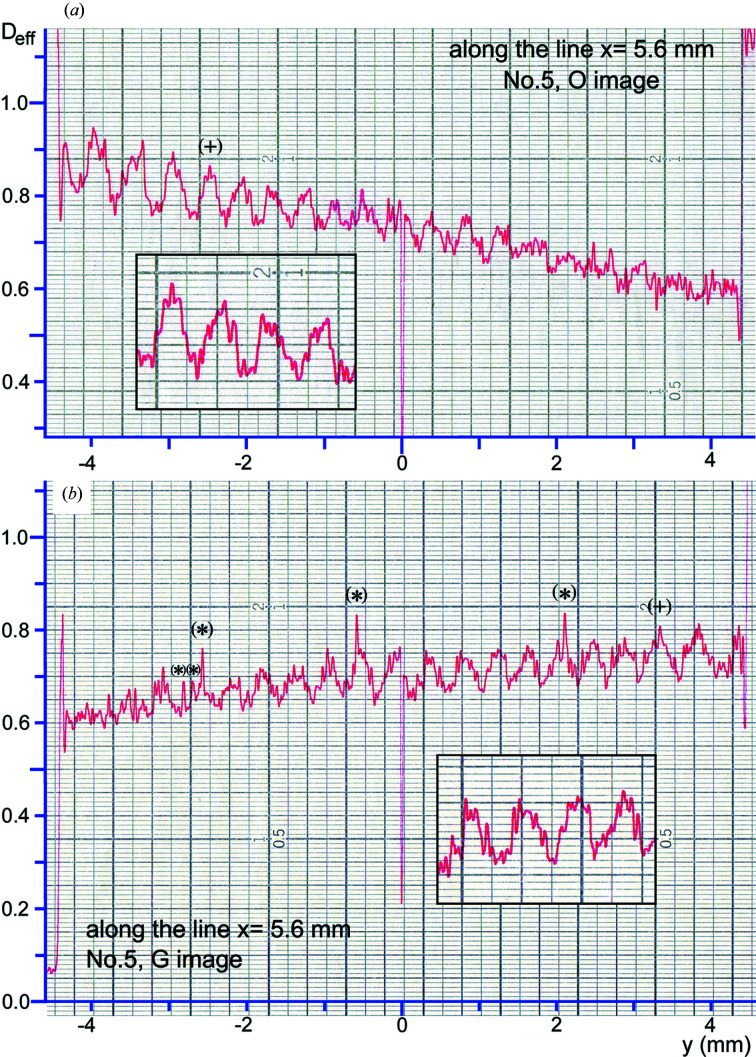
Fringe profiles measured on the experimental images in Fig. 2 ▸. Scanned at *x* = 5.6 mm along the *y* direction in the images. (*a*) Profile of the *O* image in Fig. 2 ▸(*a*). (*b*) Profile of the *G* image in Fig. 2 ▸(*b*). 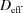 means the effective optical density in the densitometric measurement concerned. The (+) marks in (*a*) and (*b*) note pointed-top profiles as a notable peculiarity, and (*) marks in (*b*) note false intensity peaks from noises (mottles). For further details, see text.

**Figure 5:**
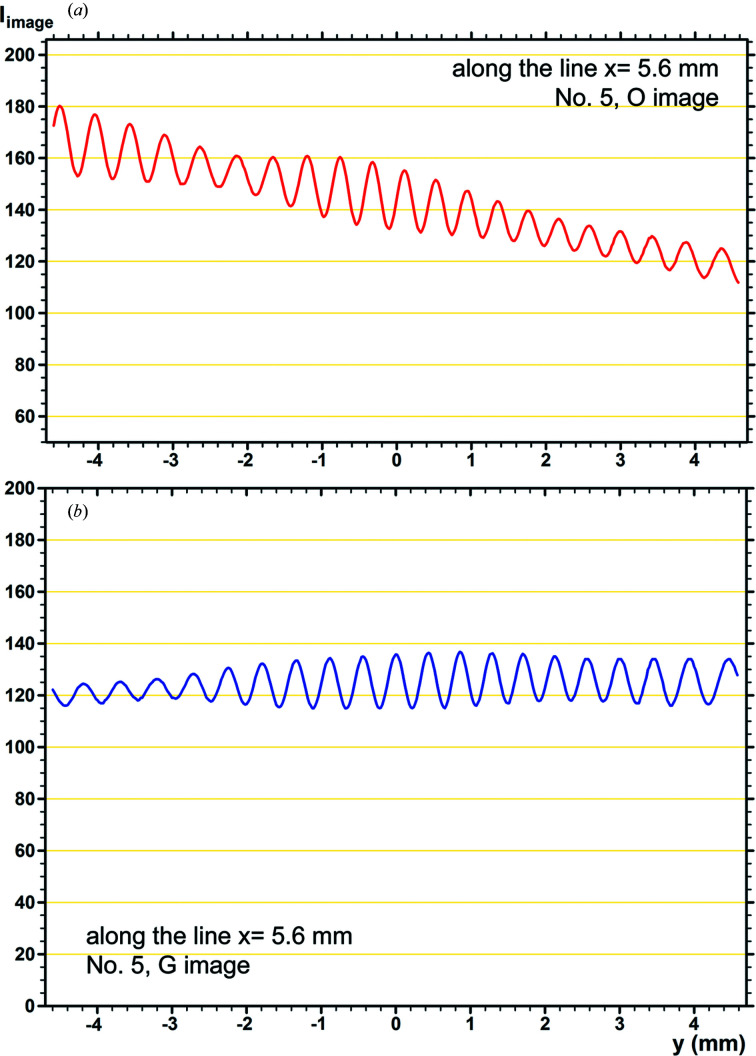
Fringe profiles measured in the simulated images in Fig. 3 ▸, corresponding to the experimental fringe profiles in Fig. 4 ▸. Scanned at *x* = 5.6 mm along the *y* direction. (*a*) Profile of the *O* image in Fig. 3 ▸(*a*). (*b*) Profile of the *G* image in Fig. 3 ▸(*b*). For details, see text.

Fig. 4 ▸ shows an example of the fringe profiles of the experimental *O* and *G* images. The fringe profiles were obtained by scanning the experimental images in Figs. 2 ▸(*a*) and 2 ▸(*b*) along the *y* direction, at a position *x* = 5.6 mm. The densitometric scan was made on the recording films with a slit size of 100 µm (*x* direction) × 20 µm (*y* direction) using a microdensitometer (Konica PDM-5, type B). Many profile charts were obtained in this measurement, by scanning the entire field of the images with an interval of 0.2 mm in the *x* direction. Here, examples of the fringe profiles measured are presented, in which the characteristics of the experimental fringe profiles are well revealed. Fig. 5 ▸ shows the corresponding fringe profiles obtained by an intensity scan on the simulated images in Figs. 3 ▸(*a*) and 3 ▸(*b*) with a scan width of 100 µm in the *x* direction.

In Fig. 4 ▸(*a*), the intensity curve is monotonically lowered towards the 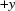 direction, aside from oscillatory modulations of the fringes. This is thought to indicate that the deviation angle increases towards the 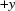 direction, in accordance with the shape of the *O*-wave rocking curve. In other words, the intensity curve above illustrates the effect of the vertical divergence of the beam, as mentioned in Section 2.2.2. Then, on the intensity curve of the *G* image in Fig. 4 ▸(*b*), a flat, maximum-intensity region which is considered to be the peak of the curve is formed within the region *y* > 2 mm. The fringe profiles in the simulated images in Fig. 5 ▸ may be assessed as simulating fairly well the general aspect of such profiles in the experimental images in Fig. 4 ▸. However, the split profile close to a PMD site shown at *y* = 2.6 mm in the experimental *O*-image profile is not successfully reproduced in the profile of the simulated image. In the profile of the *G* image in Fig. 5 ▸(*b*), the slope angle of the entire intensity curve from the 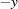 edge to the 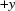 edge is rather small, compared with that in the experimental profile curve in Fig. 4 ▸(*b*). Besides, the peak of this simulated intensity curve sits at 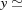 1 mm, being displaced from the presumed peak position of *y* > 2 mm in the experimental curve. These insufficient agreements were difficult to improve despite much effort.

#### 3.2.2. Origin of the coordinate in the expression of the moiré interference phase   

The positions of the fringes in the simulated image profiles in Fig. 5 ▸ agree approximately with those in the experimental image profiles in Fig. 4 ▸. (In this discussion, fringe profiles in the *G* images are mainly considered, since the experimental *O*-image profile involves a disturbance from a PMD, as mentioned above.) The approximate agreement in the fringe position was attained by adjusting the value of 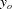 in the term 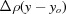 in equation (8), as 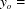 −0.26 mm. Without introducing such an adjustment, the fringe positions in the simulated image profiles remained unaligned to those in the experimental profiles. Basically, the factor to move the fringe position is 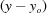, and not the deviation angle 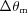 (in the case of a rotation moiré). When terms other than 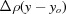 are hypothetically assumed to be zero in equation (8), 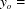 −0.26 mm is understood to indicate a position at which the two sets of crystal lattices of the component crystals coincide with each other. As a general solution it should be written as 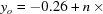 (fringe spacing) (mm). In equations (I-37*b*), (I-37*c*), the origin of the coordinate system was taken at a point of coincidence of two sets of crystal lattices. However, such a coordinate system was found to be inconvenient in treating practical moiré fringes.

The expression for the interference phase of the moiré fringes, shown in equations (I-34), (I-37) and (I-53), and in equation (8) in this paper, should be rewritten into a more general form as follows: 
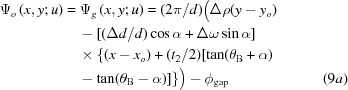


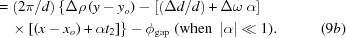
Here, (*x*, *y*) are coordinates with the origin at a general unspecified point, and (*x_o_*, *y_o_*) is a point where the two sets of crystal lattices coincide. [In light of this phase equation, the position of the lattice coincidence in equation (8) is understood to lie at (*x_o_*, *y_o_*) = (0, *y_o_*); the term 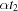 is neglected in equation (8), since 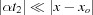.] Furthermore, the position (*x_o_*, *y_o_*) of the lattice coincidence in a strained specimen will vary with the position (*x*, *y*) in question. Therefore, a point of lattice coincidence must strictly be given as [*x_o_*(*x*, *y*), *y_o_*(*x*, *y*)]. In this simulation, all images were calculated with the coincidence site put at *y_o_* = −0.26 mm for all *x* coordinates.

#### 3.2.3. Fringe contrast   

As shown in the fringe profiles in Figs. 4 ▸ and 5 ▸, the moiré fringes under study are of considerably low contrast in the experimental as well as in the simulated images. According to the actual measurement in Figs. 4 ▸ and 5 ▸, fringe contrast in the experimental *O* and *G* images is *V* = 5.7% and *V* = 5.8%, respectively, in the mean, and that in the simulated *O* and *G* images is *V* = 6.0% and *V* = 6.5%, respectively. The contrast values were calculated as *V* = 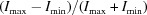, with 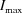 and 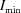 being the maximum and minimum intensities, respectively. The effective optical density 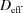, with which the ordinate axis in the experimental profiles is graduated, is understood to be in proportion to the image intensity, since the optical density is not high (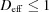) in the present case [specifically, the relationship 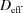 = 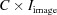 holds, *C* and 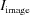 being an appropriate proportional constant and the image intensity equal to 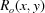 or 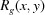, respectively]. The low fringe contrast is a speciality of the present experimental moiré images. As shown in Paper II, the low fringe contrast is considered to be due to the inter­spacing gap in the specimen bicrystal; the gap widths are estimated to be 243 µm, or assumed to be 234 µm, for the experimental and simulated images, as stated in the preceding sections. The good agreement shown in the fringe contrast between the experimental and simulated images should be noted.

#### 3.2.4. Fringe profiles of singular shapes in the experimental moiré images   

Unlike the approximate agreement in the fringe contrast described above, noteworthy differences between the experimental and simulated images are clearly recognized in the shapes of fringe profiles. The shapes of the fringe profiles of the simulated images in Figs. 5 ▸(*a*) and 5 ▸(*b*) do not significantly deviate from a symmetric shape, although some of the profiles are somewhat asymmetric. In addition, profile shapes do not abruptly change between neighbouring fringes. Such characteristics may be considered to be an ordinary aspect of the intensity profile obtained from the calculation of a smooth function. On the other hand, in the entire field of the experimental images in Figs. 4 ▸(*a*) and 4 ▸(*b*), fringe profiles with a strongly asymmetric shape are commonly observed, and the direction of asymmetry often switches abruptly between neighbouring fringes. Typical examples of such asymmetric fringe profiles are shown in a magnified scale in the insets in Figs. 4 ▸(*a*), 4 ▸(*b*). In addition, profiles with a pointed top, as noted by the (+) mark, were occasionally observed, though not too frequently. Such profiles with pointed tops have a common feature that their shapes are nearly symmetric on the whole. A theoretical derivation of such singular-shaped fringe profiles is difficult to achieve based on the present moiré-fringe theory, as is easily seen, while many other features of the experimental moiré images have been successfully explained by the same theory. To solve this problem, it would be necessary to know the reason for the singular shapes of the fringe profiles and to construct a new higher-level handling theory.

## 4. Results of computer simulations II   

### 4.1. Moiré images of two crystals inclined to each other   

#### 4.1.1. Moiré images obtained by experiment and simulation   

**Figure 6:**
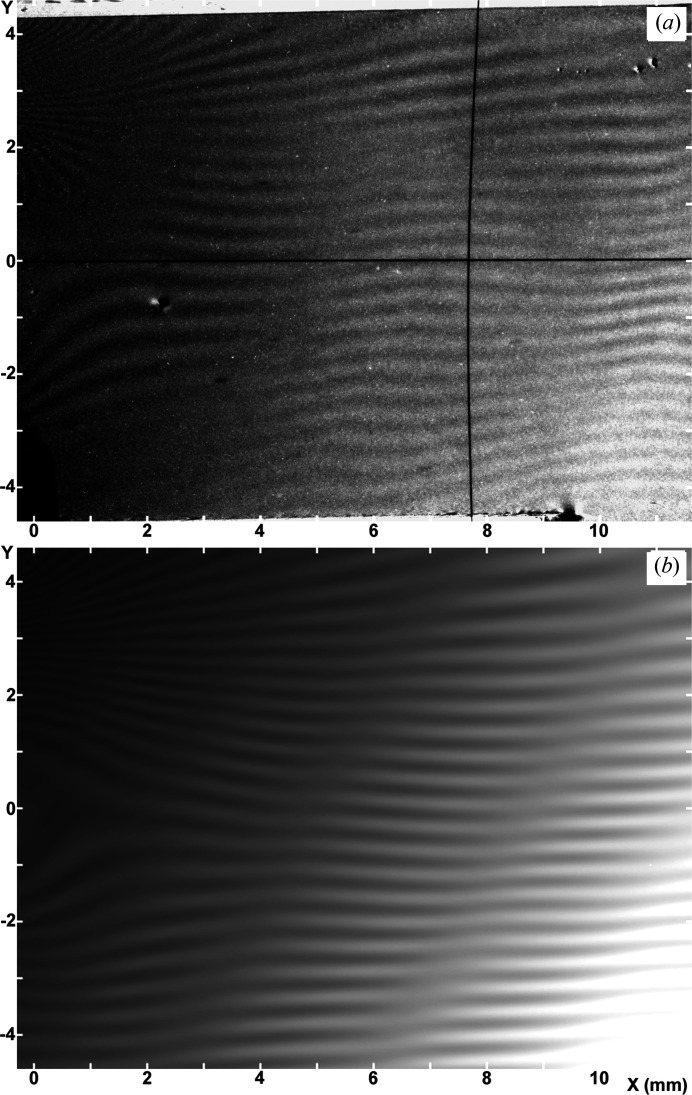
(*a*) Experimental (T set No. 8) and (*b*) computer-simulated moiré images when the rear component crystal was forcedly rotated about the *y* axis towards the front crystal. *O* image, Si 220 reflection, λ = 0.072 nm. 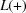 = 0.2 gf (= 1.96 mN) and 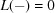. For further explanation, see text and Table 3 ▸.

**Figure 7:**
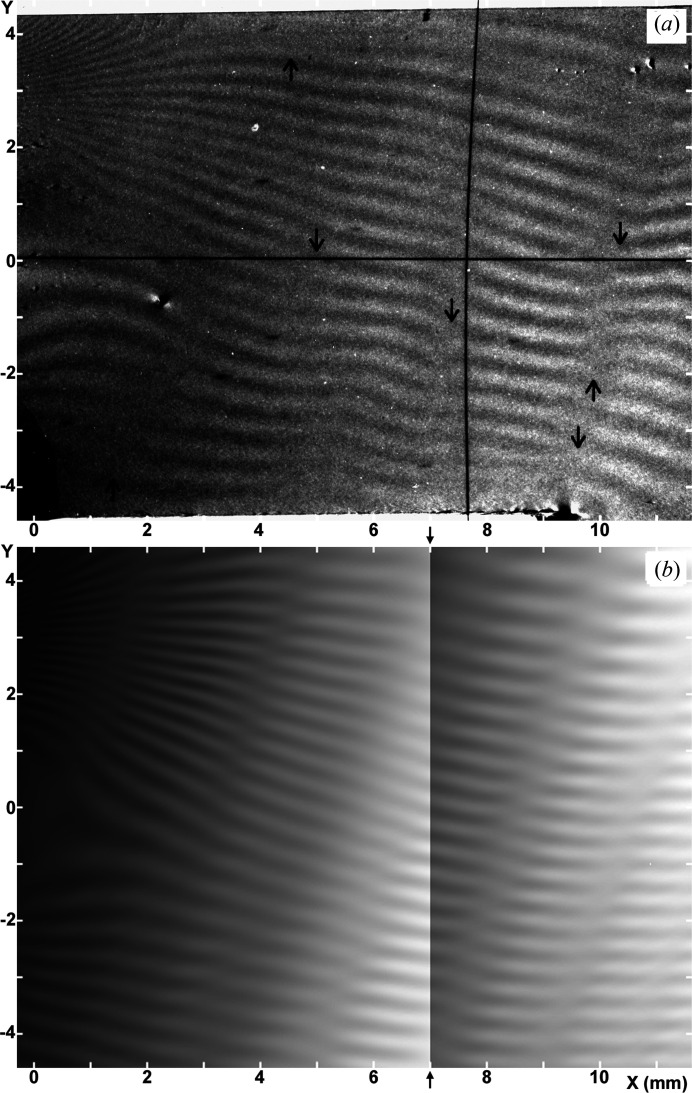
(*a*) Experimental (T set No. 14) and (*b*) computer-simulated moiré images when the rear component crystal was forcedly rotated about the *y* axis towards the opposite side of the front crystal. *O* image, Si 220 reflection, λ = 0.072 nm. 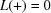 and 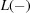 = 0.2 gf (= 1.96 mN). The brightness in the simulated image (*b*) was adjusted by dividing the entire field of the image into two partial fields, since it was difficult to suitably adjust it within 256 graduations in the one entire field. For further explanation, see text and Table 3 ▸.

Figs. 6 ▸(*a*) and 7 ▸(*a*) show experimental moiré images (*O* image) taken when the rear crystal of the bicrystal was forcedly rotated by a minute angle about the *y* axis. The force applied to cause the rotation was 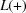 = 0.2 gf (= 1.96 mN) for the image in Fig. 6 ▸(*a*), and 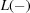 = 0.2 gf for the one in Fig. 7 ▸(*a*) (see Fig. 1 ▸). Compared with the moiré image in Fig. 2 ▸(*a*) taken with no forced rotation, the fringes slope towards the upper-right direction [Fig. 6 ▸(*a*)], or towards the lower-right direction [Fig. 7 ▸(*a*)]. Although it is not concerned with the present discussion on the forced-rotation effect, Fig. 6 ▸(*a*) is the moiré image which has been shown as the main data of the moiré-image experiment in question (Yoshimura, 1996*a* ▸, 1997*c* ▸). A motive for doing such an experiment with a forcedly rotated crystal was a discussion on the moiré pattern when the two crystals concerned are inclined to each other (Hashimoto *et al.*, 1961 ▸; Nagakura, 1972 ▸). Obtaining the results as shown in Figs. 6 ▸(*a*) and 7 ▸(*a*), the author for a while had considered that the effect of the inclination of the lattice plane on the moiré pattern, namely the effect of 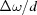 as the third component of 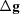, was evidenced. However, this understanding was contradicted by a subsequent theoretical consideration (Yoshimura, 1997*b* ▸), which shows that the effect of the lattice-plane inclination on the moiré-interference phase is 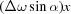 [see equation (8)], and is impossible in symmetric Laue geometry (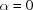). Since then, it had been a question of why the fringe pattern was changed with the 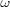 rotation, despite a theoretical indication otherwise. During repeated simulations in the present work, it came to be found that the angle α for producing sloped moiré fringes need not be so large, and an angle of 0.5° or so suffices to make an appreciable fringe slope, in this case of nearly rotation-moiré fringes of Λ ≃ 0.44 mm. The possibility of a misorientation of the inner crystal surfaces of such an order of magnitude was not excluded in the experiment under discussion.

As mentioned earlier in Section 2.1.2, a misorientation by α = −0.37° from the exact (111) orientation was actually found in the inner surfaces of the bicrystal. After a time-consuming computational study with the value 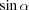 = −0.00646 put into the phase functions 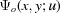 and 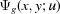 in equation (8), conclusive simulated images were obtained as shown in Figs. 6 ▸(*b*) and 7 ▸(*b*). The parameters for the simulated images are given in Table 3 ▸. In this case also, the values of 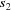 and 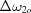 were determined with main attention paid to the positions, inclinations and spacings of LC bands, analogously to the preceding case of Fig. 3 ▸. In addition, in this case, attention was also paid to the running direction of moiré fringes. The values of 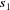 and 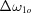 were taken to be the same as in the case of Fig. 3 ▸, since no change should have occurred to the state of the front crystal by the experiment operation (forced rotation) in question. The values of the mid-deviation angle 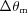 were adjusted through the observation of the entire view of simulated images, including the aspect of LC band patterns, the image intensity distributions *etc*. The values of 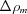 (= −*d*/0.44 mm) and 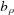 (0.003 mm^−2^), and the LEC local strains 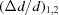 and 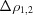 were the same as in the case of Fig. 3 ▸.

It can be confirmed that the magnitudes of 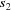 and 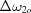 in Tables 1 ▸ and 3 ▸ are in the order 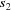 [Fig. 6 ▸(*b*)] < 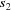 [Figs. 3 ▸(*a*), 3 ▸(*b*)] < 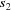 [Fig. 7 ▸(*b*)] and 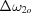 [Fig. 6 ▸(*b*)] > 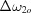 [Figs. 3 ▸(*a*), 3 ▸(*b*)] > 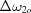 [Fig. 7 ▸(*b*)], being consistent with the operation of the forced rotation to the rear crystal in a qualitative sense. The inclined running directions of moiré fringes in Figs. 6 ▸(*b*) and 7 ▸(*b*) agree approximately with those in the corresponding experimental images. The number of fringes over the entire *y* range in the simulated images agrees well with that in the experimental images with a difference of less than one fringe, except for regions 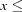 1 mm and 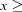
9 mm in the case of Fig. 6 ▸(*b*), and regions 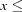 1 mm and 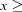
10 mm in the case of Fig. 7 ▸(*b*). From these observations, Figs. 6 ▸(*b*) and 7 ▸(*b*) may be assessed to simulate fairly well the experimental images in Fig. 6 ▸(*a*) or Fig. 7 ▸(*a*).

However, it should be commented that the simulation of the LC band patterns is not good enough, particularly in Fig. 6 ▸(*b*), although the images in Figs. 6 ▸(*b*) and 7 ▸(*b*) were the best attainable results. Similarity in the band pattern, which worsens in the upper region of the images, would probably be related to a non-uniformity in the bend and rotation of the crystal plate, due to application of the force 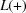 or 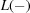 at an offset position on the crystal edge [see Fig. 1 ▸(*b*)].

#### 4.1.2. Analysis of the slope of the fringe lines   

Moiré fringes are generally inclined at −3°–+9° to the *x* axis, mainly in the positive-angle direction, in the area 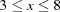 (mm) in the image in Fig. 6 ▸(*b*), and are inclined at −18°–−10° to the *x* axis in the same area in Fig. 7 ▸(*b*). In the image in Fig. 3 ▸(*a*) the corresponding slope angle is −10°–+6°; these slope angles were manually measured on the images. According to the phase equation in equation (8) the slope of fringes is estimated by 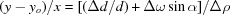. However, it was difficult to explain the fringe slopes satisfactorily by this estimation only, in the present case. According to the elasticity calculation in equation (10*a*), the value of 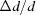 is −2.8 × 10^−8^ to +7.3 × 10^−8^ within the entire *y* range [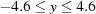 (mm)], and is +4.4 × 10^−8^ at *y* = 0, when estimated at *x* = 6.0 mm. This 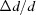 value is unchanged throughout the computations of Figs. 3 ▸(*a*), 3 ▸(*b*) and Figs. 6 ▸(*b*), 7 ▸(*b*). The angle 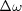 {= 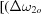 − 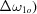 + (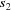 − 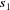) (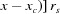}, on the other hand, is changed with the images concerned. It is given in arcseconds as 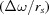 = 0.23 + 0.030*x* in the case of Fig. 6 ▸(*b*), and 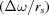 = −1.08 + 0.055*x* for Fig. 7 ▸(*b*), using the 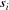 and 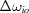 values (*i* = 1, 2) in Table 3 ▸; for the image in Fig. 3 ▸(*a*), 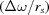 = −0.26 + 0.049*x*. For these (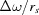), the value of 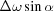 is −1.3 × 10^−8^ and 2.3 × 10^−8^ (rad) at *x* = 6.0 in the cases of Figs. 6 ▸(*b*) and 7 ▸(*b*), respectively. The corresponding fringe slopes are roughly estimated to be −4° and −9° to the *x* axis, respectively, with 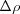 assumed to be −*d*/0.44 mm. These angles deviate significantly from the actually observed fringe slopes.

Table 4 ▸ shows the change in the slope angle of moiré fringes and that in the (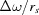) angle in Figs. 6 ▸(*b*) and 7 ▸(*b*), relative to the angles of the fringe slope and the 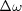 inclination in Fig. 3 ▸(*a*). From a roughly good correspondence between the compared angular values of the fringe slope and (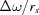), shown in Table 4 ▸, it may be seen at a semiquantitative level that the change in the fringe slope in Figs. 6 ▸(*b*) and 7 ▸(*b*) is related to the change in the (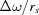) angle. Based on the agreement between the simulated and experimental images mentioned in Section 4.1.1, the change in the fringe slope in the experimental images in Figs. 6 ▸(*a*) and 7 ▸(*a*), relative to the fringe slope in Fig. 2 ▸(*a*), may also be understood in the same way as described above for the simulated images.

If we want to understand more fully the fringe slope in the simulated and experimental images, we have to take into account the influences of the gap phase 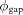 and the *Pendellösung* oscillation phase, as mentioned in Sections 3.2.2 and 3.2.3 in Paper II. The large-angle slope of the fringes in Figs. 7 ▸(*a*) and 7 ▸(*b*) is understood as being mainly produced as a pattern of obliquely extending fringes (see Section 3.2.2 in Paper II), although the fringe slope due to the term 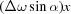 contributes additively to this large-angle slope. However, an explanation taking these two additional phases into account is not a simple task, and will inevitably be lengthy. Furthermore, occurrence of the LEC local strains, which are involved in the estimation of 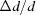 and 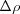, and vary with the position (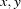), makes the fringe analysis further complicated. In this paper, the discussion of the effect of 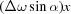 on the fringe slope finishes here.

### 4.2. Wide-area survey of moiré images diffracted from a large curved bicrystal   

#### 4.2.1. Broad-band images of a curved bicrystal   

**Figure 8:**
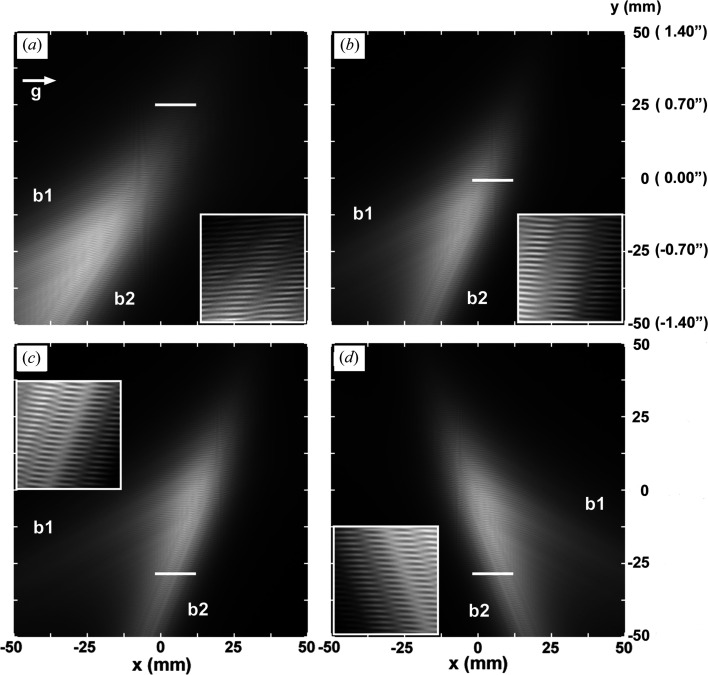
Wide-area diffraction moiré images (*G* images) from a curved bicrystal, computed for a better and unified understanding of the simulated and experimental moiré images in Figs. 2 ▸, 3 ▸, 6 ▸ and 7 ▸. For further explanation, see text.

To better understand the images shown in Figs. 2 ▸, 3 ▸, 6 ▸ and 7 ▸, computed wide-area moiré images (*G* image) are shown in Fig. 8 ▸, which are imaginarily assumed to be diffracted from a large curved bicrystal with the incidence of a laterally wide and vertically divergent X-ray beam. Curvatures of the front and rear crystals are assumed to occur about the vertical axis (*y* axis) in the same way as in Figs. 2 ▸, 3 ▸ *etc*. The assumed arrangement for computing diffracted images is the same as for the images in Figs. 2 ▸, 3 ▸ *etc*., and the diffraction vector 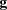 is directed from left to right in the horizontal direction, as shown in Fig. 8 ▸(*a*). The two fuzzy streak- or band-like images marked with *b*1 and *b*2 in Figs. 8 ▸(*a*)–8 ▸(*d*) are partial images of strong diffraction intensity in the entire wide-area bicrystal images, being related to the front and rear crystals, respectively. They are hereafter referred to as broad-band images, being distinct from the low-contrast band images mentioned previously. Along the abscissa axis in each figure, the *x* coordinate is given with respect to the wide-area bicrystal image. The values of (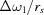) and (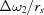) vary along this axis, relating to the curvatures in the crystals. Strong diffraction occurs in a limited range in (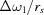) or (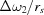), namely within a limited range of *x*, and the region of strong intensity giving a broad-band image moves along the *x* axis with the change in (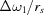) and/or (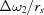). Along the ordinate axes in the figures, the *y* coordinate in the wide-area bicrystal image is graduated. The angular variation 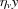 (arcseconds) of the vertical divergence of the beam and the 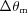 angle for indicating the angular positions of the images in the insets are also graduated along this axis; the values in parentheses behind the *y* values give the corresponding angular graduations.

The wide-area bicrystal image in each figure was computed for the mid-deviation angle of 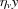, with 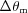 = 0.0′′. The angular width of the incident beam was 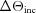 = 0.34′, as in the previous computations. It was assumed that 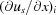 = 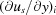 = 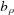 = 0, regarding the LEC local strains and the strain of elastic bend about the *z* axis. The width and extending directions of the broad bands *b*1 and *b*2 depend on the strength and sign of the curvatures 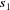 and 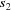. The broad-band images are horizontal (parallel to the *x* axis) when 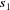 = 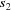 = 0, and the slope of their extending direction from the *x* axis increases with the value of 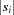 (*i* = 1, 2). As seen from a simple consideration of the diffraction geometry for a curved crystal, a broad-band image slopes towards the upper-right direction when 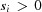, and slopes towards the lower-right when 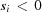. Images (*a*)–(*c*) correspond to the images in Fig. 6 ▸(*b*), Figs. 3 ▸(*a*), 3 ▸(*b*) and Fig. 7 ▸(*b*), respectively, which have been shown earlier. Although the parameters for computing images (*a*)–(*c*) are the same as those in Tables 1 ▸ and 3 ▸, they are mentioned again: image (*a*) *s*_2_ = 0.045′′ mm^−1^, 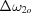 = 0.54′′; image (*b*) *s*_2_ = 0.064′′ mm^−1^, 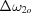 = 0.27′′; image (*c*) *s*_2_ = 0.070′′ mm^−1^, 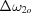 = −0.50′′. Image (*d*) is computed with *s*_1_ = −0.015′′ mm^−1^, *s*_2_ = −0.070′′ mm^−1^, 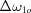 = −0.10′′ and 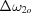 = −1.00′′. However, images (*a*)–(*d*) in this Fig. 8 ▸ are all *G* images unlike Figs. 6 ▸(*b*) and 7 ▸(*b*) of *O* images. [Furthermore 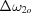 = 0.54′′, for Fig. 8 ▸(*a*) slightly disagrees with 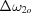 = 0.57′ for Fig. 6 ▸(*b*).] The image with 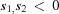 in Fig. 8 ▸(*d*) is added for reference to the images with 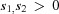 in Figs. 8 ▸(*a*)–(*c*).

#### 4.2.2. Consistency in the simulations of different experimental images   

In Figs. 8 ▸(*a*)–8 ▸(*d*), the inset shows an enlarge­ment of a local region in the broad-band images, at the angular positions indicated by the white lines. The inset image was computed under the same conditions as the matrix broad-band image, but at an enlarged scale and with the mid-deviation angle of 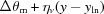. Then, 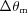 indicates an angular position on the ordinate axis, at which height the white line is drawn, 0.70′′, −0.03′′, −0.80′′ and −0.80′′ in (*a*)–(*d*), respectively; 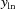 gives the position at the white lines. In Figs. 8 ▸(*a*) and 8 ▸(*c*), for the images in Figs. 6 ▸(*b*) and 7 ▸(*b*), respectively, the local regions from which the inset images were sampled lie away from the intersecting region of the two broad bands, and the intensity of broad-band image *b*1 declines significantly there. Low-contrast bands seen in the inset images run almost parallel to the extending direction of broad-band image *b*2, suggesting that the intensity of broad-band image *b*2 is dominant in the total image intensity. Nevertheless, moiré fringes appearing with good contrast in the inset images suggest that the broad-band image *b*1 also has a low but significant intensity there, taking part in the formation of the moiré fringes. Unlike these cases, the local region sampled for the inset image in Fig. 8 ▸(*b*), for the images in Fig. 3 ▸(*b*), lies close to the intersecting region of the two broad bands (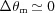), where they have mutually comparable intensities. Presumably in connection with such a condition, the low-contrast bands in the inset image stand more upright than in the other two cases.

Based on the understanding of the characteristics of the curved bicrystal diffracted images, described above, the choice of positive values of 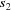 for both Figs. 6 ▸(*b*) and 7 ▸(*b*) is confirmed to be certainly correct, from the extending direction of the low-contrast bands in the experimental images in Figs. 6 ▸(*a*) and 7 ▸(*a*). If 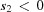, the low-contrast bands should lean towards the opposite side, as shown in Fig. 8 ▸(*d*). Regarding the sign of curvature 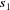, no clue is obtained about it. However, when assuming 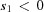 under the use of positive values of 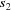, no good simulated images could be obtained despite many attempts made by changing the values of 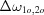, 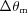 *etc*. From this result, the positive value *s*_1_ = 0.015′′ mm^−1^, as mentioned already, is considered to be correctly evaluated. The direction and spacing of low-contrast bands in the inset images in Figs. 8 ▸(*a*)–8 ▸(*c*) agree approximately with those in the corresponding images in Figs. 6 ▸(*b*), 3 ▸(*b*) and 7 ▸(*b*), and thus the inset images are a good substitute for the simulated images. From the perspective of the entire wide-area bicrystal images in Figs. 8 ▸(*a*)–8 ▸(*c*), with the inset images positioned on the respective white lines in the wide-area images, it can be seen that the simulated images, *i.e*. the solutions of the simulation study, are obtained with consistency through the assumed experiment operation of the forced rotation of the rear component crystal. In other words, it can be seen there that, with the change in the forced-rotation load from 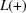 = 0.2 gf to 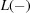 = 0.2 gf through the intermediate unloaded state, 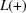 = 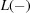 = 0, the aspect of the wide-area bicrystal image including the inset image changes in an understandable way, from the aspect in (*a*) to that in (*b*), and from the aspect in (*b*) to that as in (*c*). The mentioned consistency among the simulated images guarantees that the computation of the simulated images and the related characterization of the corresponding experimental images are certainly correct.

## 5. Conclusions and supplementary remarks   

The present paper concludes with the following remarks:

(i) The theoretical computations of the moiré images and fringe profiles shown in Figs. 3 ▸, 5 ▸, 6 ▸(*b*) and 7 ▸(*b*) show, on the whole, satisfactory simulations for the experimental moiré images and fringe profiles which are the subject of this simulation work, although some partial aspects in the experimental images remain unsatisfactorily simulated in part. Serious disagreement between the simulated and experimental images suggesting a problem in the employed theory was not found, with respect to the study of the fringe-and-band patterns in the images. Partial insufficiencies in the simulations for the image in Fig. 2 ▸(*a*) (*O* image) *etc*. and for the fringe profiles in Figs. 4 ▸(*a*) and 4 ▸(*b*) [aside from the problem described in remark (iii) in this Section] are thought to be largely due to the insufficiency in the estimation of the LEC local strains and other strains in the specimen crystal, and due to the disregard of the small variations in the crystal thicknesses, as mentioned in Section 2.1.2. To conclude, this simulation study had basically correct consequences for the simulation of the previous experimental moiré images in question. Conversely, this moiré-fringe theory was verified to be correct through a check of the experimental images. One more important factor in the successful simulation was the success in the theoretical analysis of the LEC local strains.

Furthermore, the necessity to pay attention to the vertical divergence effect of the beam in synchrotron X-ray diffraction has been described through practical examples of experimental moiré topographs and their theoretical simulations (Section 2.2.2). It was found that in the analysis of the moiré fringes of a crystal having an inhomogeneous strain, the fringe spacing in some cases is determined in a different way from the simple estimation using the formula 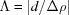 or 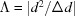 (Section 3.1.3). In the simulation of the intensity profile of moiré fringes, it was shown that the coordinate origin (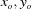) in the calculation of the interference phase needs to be introduced, so that fringe positions in the simulated images agree exactly with those in the experimental images (Section 3.2.2).

(ii) It should be noted that the experimental images in Figs. 6 ▸(*a*) and 7 ▸(*a*) were reproduced almost satisfactorily in the respective simulated images, which were computed as moiré images when the two component crystals of a bicrystal are inclined towards each other by a small angle 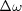 about the *y* axis. Based on this successful simulation, it was mostly confirmed that the observed changes in the fringe slope in the experimental moiré images were caused by the induced lattice-plane inclination 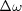 as just mentioned. These would probably be the first examples of moiré images in which the lattice-plane inclination 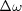 is seen to take part as an element of the third component of 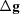, when 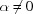. However, in this paper, the effect of 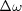 on the fringe slopes could not be shown very accurately, due to complications in the analysis procedure. In view of the significance of the problem, an accurate confirmation of the effect of 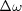 should be carried out, by conducting an additional experiment using an unstrained crystal.

(iii) This simulation work was also the first attempt at a full theoretical explanation for the previous experimental moiré images in question. Through the good theoretical simulation for the experimental images, as mentioned in remark (i), the attempted explanation was made to an almost satisfactory level, with respect to the fringe-and-band patterns of the images.

**Figure 9:**
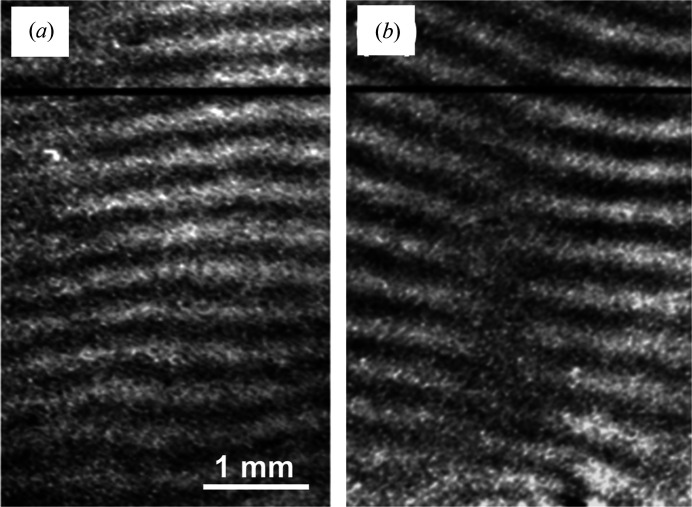
Experimental moiré images showing fine subsidiary fringes, taken in the same experiment as mentioned elsewhere in this paper. (*a*) *O* image. The same image as in Fig. 6 ▸(*a*), but reproduced at a higher magnification. (*b*) *G* image. Taken simultaneously under the same condition with the *O* image in Fig. 7 ▸(*a*).

**Figure 10:**
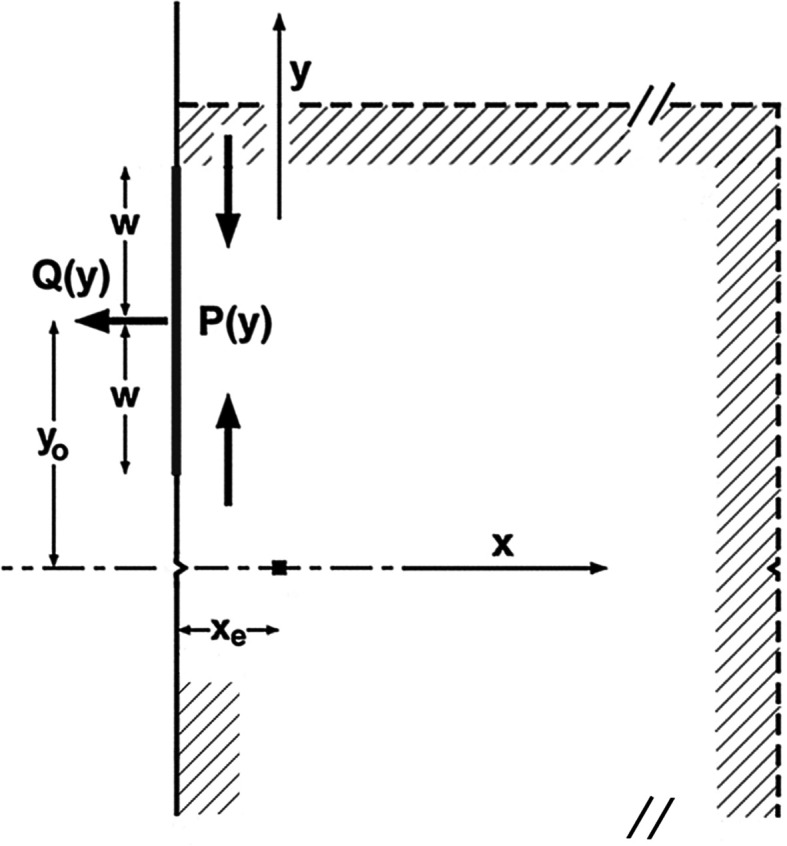
Model of the strain fields around the left edge of the specimen crystal, for the elasticity calculation of the LEC local strain in Appendix *A* [App appa]. Dashed lines indicate the edge of the specimen crystal. 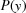, contraction stress due to gluing [ = 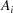 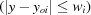; = 0 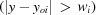]. 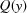, pulling stress from a balancer weight [ = 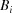 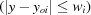; = 0 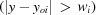].

However, peculiar features of fringe profiles such as strongly asymmetric fringe profiles and pointed-top profiles, as shown in Figs. 4 ▸(*a*) and 4 ▸(*b*), could not be simulated, despite the success in the simulation of the global features of the images. Fringe profiles of such peculiar shapes are not particularly special in the experiment under discussion, and are commonly observed in any image. In addition to such peculiarities of the fringe profiles, another noteworthy finding is the occurrence of fine subsidiary fringes as shown in Fig. 9 ▸ (Yoshimura *et al.*, 2001 ▸; Yoshimura & Hirano, 2009 ▸). Superposed on the main fringes that give the moiré pattern, they are observed with a very weak contrast, to run along a direction crossing the main fringes at a high angle. Such subsidiary fringes were also commonly observed in any moiré image in the experiment under study. If due attention is given, they can be recognized in any of the images in Figs. 2 ▸(*a*), 2 ▸(*b*) and Figs. 6 ▸(*a*), 7 ▸(*a*), although with much worse visibility than in Fig. 9 ▸. Although a presentation of clear subsidiary fringes on printed papers is not easy in general, those in Fig. 9 ▸ were presented with somewhat good visibility, owing to their original good contrast and to a special contrast-enhancement treatment. The cause and generating mechanism of such fine subsidiary fringes is not known. However, they should normally also be considered to be a record of the wavefield in the imaging experiment. The peculiar-shaped fringe profiles and occurrence of fine subsidiary fringes, as described above, seem to be beyond the treatable limit of the presented moiré-fringe theory. The addition of some new elements to the theoretical basics of the X-ray diffraction optics seems to be needed.

(iv) To add more in connection with the problems mentioned in remark (iii), one of the critical comments given thus far on the experimental images in question was that the result may be brought about by noise, and is unreliable unless they were recorded on nuclear plates. The author initially could not understand the meaning of ‘noise’, but what it means has been suggested in later experiences. Use of X-ray films and developing and fixing solutions results in the occurrence of many fine mottles on the films, unless special attention is paid in the film processing. Such mottles may be referred to as noise in a detailed study of fringe profiles. However, using X-ray films and processing solutions of the same brand name (Fuji type No. 50) at the time of the experiment, without any special attention given to the developing and fixing, not many mottles occurred. Although the occurrence of mottles was not completely suppressed, they were very few as can be confirmed in Figs. 2 ▸(*a*), 2 ▸(*b*) and Figs. 6 ▸(*a*), 7 ▸(*a*), and do not have a significant influence on the study of fringe profiles.

Another critical comment was that the described peculiarities or abnormalities of the images were not confirmed in reexaminations by other researchers. To answer this comment, the author would like to draw attention to the fact that the moiré images in question were taken under the incidence of a quasi-plane wave with an angular width of 0.34′′, and are of considerably low fringe contrast (see Section 3.2.3). Furthermore, the thinness of the film emulsion layer recording the images, which was as thin as 10 µm, also would possibly have had some effect on the easy notice of the abnormalities. Though not studied sufficiently, it has been observed that peculiar-shaped fringe profiles and the presence of fine subsidiary fringes become less noticeable with an increase in the angular width of the incident wave. Possibly, these abnormalities would not practically be observed in Lang topography. Besides, when the fringe contrast is enhanced, the abnormalities become less observable. If the experiment is conducted under similar conditions to the previous reports (Yoshimura, 1993 ▸, 1996*a* ▸), with attention given to the points mentioned above, similar results to those mentioned herein should be obtained by any experimenter.

## Supplementary Material

Derivation of equations (10a)-(10d) in Appendix A. DOI: 10.1107/S205327332000532X/td5063sup1.pdf

## Figures and Tables

**Table 1:** Parameters used for computing simulated images. (I)

	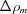	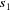	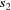	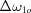	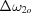	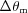
Figs. 3 ▸(*a*), 3 ▸(*b*)	−0.090′′	0.015′′ mm^−1^	0.064′′ mm^−1^	0.06′′	0.27′′	−0.03′′

**Table 2:** Detailed comparison of fringe spacings in the upper-left corner area [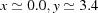 (mm)] in the simulated [Fig. 3 ▸(*b*)] and experimental [Fig. 2 ▸(*b*)] moiré images Comparison between the two cases when 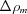 = −0.44 × 10^−6^ (rad) is assumed and when 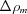 = +0.44 × 10^−6^ (rad) is assumed. 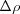 = 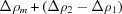. 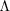 (simul.) and 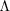 (exp.) denote the actually observed fringe spacings in the simulated and experimental images, respectively.

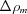 (×10^−6^)	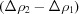 (×10^−6^)	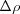 (×10^−6^)	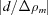 (mm)	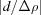 (mm)	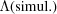 (mm)	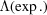 (mm)
−0.44	−0.25	−0.69	0.44	0.28	≃0.14	≃0.10
0.44	−0.25	0.19	0.44	1.03		

**Table 3:** Parameters used for computing simulated images. (II)

	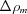	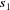	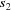	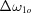	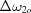	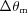
Fig. 6 ▸(*b*)	−0.090′′	0.015′′ mm^−1^	0.045′′ mm^−1^	0.06′′	0.57′′	0.70′′
Fig. 7 ▸(*b*)	−0.090′′	0.015′′ mm^−1^	0.070′′ mm^−1^	0.06′′	−0.50′′	−0.80′′

**Table 4:** Change in the slope angle of moiré fringes and that in the 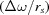 angle, relative to the angle of the fringe slope and 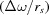 in Fig. 3 ▸(*a*)

	Fig. 6 ▸(*b*)	Fig. 7 ▸(*b*)
Change in the fringe-slope angles	+3°–+7°	−16°–−8°
Change in the 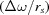 angles (at *x* = 6.0 mm)	+0.38′′	−0.78′′

## Appendix A Analytical expressions of strain distributions induced by stresses on the left edges of the specimen bicrystal

The solution of analytical expressions of the strain distributions could be found by applying an exercise of elasticity theory (Takeuchi, 1969 ▸; Exercise No. 64) to the problem. Here only the results of the calculation are shown; the process of the calculation is described in the supporting information.

From the expressions of the displacements of strain given in equations (S6*a*) and (S6*b*) in the supporting information, expressions of the components of strain are obtained as follows:

Here,  and  denote the displacement in the *x* and *y* directions, respectively;  and  represent the strengths of contraction (in the *y* direction) and pulling (in the *x* direction) stresses, respectively (see Fig. 10 ▸); the index *i*, attached to the expressions of strains  *etc*. and to the constants ,  and other symbols, indicates a reference to the component crystal considered;  refers to the front crystal and  to the rear crystal. Symbols  and  denote Lamé’s constants, which were taken to be  = 0.54 × 10^11^ Pa and  = 0.70 × 10^11^ Pa. The coordinate  is that of the left edges of the component crystals, and was set to be  = −1.6 mm;  and  indicate the middle position in the range where stresses  and  work, and the width of the stressed range, respectively.

The strengths of stresses  were determined by comparing the simulated images computed for various assumed values of  with the standard experimental image [Fig. 2 ▸(*b*)]. The values with which the computed image best fits the experimental image were approximately as follows: *A*_1_ = −260 000 Pa and *A*_2_ = −60 000 Pa.  was estimated to be 478 Pa, corresponding to the balancer weight 0.236 gf. For , no balancer weight was attached on the rear-crystal edge, and therefore . The values of  and  were determined also from the inspection of the specimen, and by seeing the degree of fit of the simulated images with the experimental image. As a conclusion, they were determined to be  = 2.4,  = −2.3,  = 1.8 and  = 0.5 (in mm).

## Appendix B List of symbols

▸

**Table:**

	Position vector on the exit surface of the rear component crystal.
	Rectangular coordinates applied for the specimen crystal and for presented moiré images by experiment and in theoretical simulation [].
	*x* coordinate at which the local inclinations of the diffraction plane due to curvatures and become zero (taken to be = 9.5 mm).
	*y* coordinate at which the moiré phase becomes zero, generally not coincident with the general coordinate origin.
	*y* coordinate of the starting point of a bending or a torsional rotation of the crystal plates (assumed to be = −4.7 mm).
	Middle position of the stress distribution of the LEC local strain on the left edges of the front (*i* = 1) or rear (*i* = 2) crystals.
	Thicknesses of the front (*i* = 1) or rear (*i* = 2) component crystals.
	Width of the interspacing gap between the front and rear component crystals
;	Relative variation in the lattice spacing in the front (*i* = 1) or rear (*i* = 2) crystals, caused by LEC local strain; .
	Relative rotation about the *z* axis between the front and rear component crystals, which is responsible for the rotation-moiré pattern.
	Minute rotation about the *z* axis by gravity in the front (*i* = 1) or rear (*i* = 2) crystals; .
	Added rotation of the lattice plane about the *z* axis in the front (*i* = 1) or rear (*i* = 2) crystals, caused by LEC local strain.
	Correction factor to , relating to the weak bending in the component crystals.
	Inclination of the diffracting lattice plane about the *y* axis in the front (*i* = 1) or rear (*i* = 2) crystals; = .
	Positionally invariable part in or .
	Strengths of the curvatures (arcseconds mm^−1^) of the diffraction plane about the *y* axis in the front (*i* = 1) or rear (*i* = 2) crystals.
	Pulling weight for causing a forced rotation of the rear component crystal.
	Torsional rotation of the diffracting plane about the *y* axis, presumed from the 2.4° tilt of the component crystals.
	Conversion factor ( = 4.8481 × 10^−6^) from arcseconds to radians.
	Tilt angle of the orientation of the interspacing gap surfaces from the exact (111) orientation (measured to be α = −0.37° = −0.00646 rad).
	Middle wavelength (= 0.072 nm) in the wavelength spread of the incident beam.
*K*	Wavenumber ().
*d*	Lattice spacing of the diffracting lattice plane.
	Reciprocal-lattice-vector difference between the front and rear crystals of the specimen crystal, to produce moiré fringes (for detail, see Paper I).
	Incidence glancing angle to the diffracting lattice plane.
	Bragg angle.
	Deviation angle of the incident beam from the exact Bragg angle; .
	Deviation angles from the exact Bragg angle at, respectively, the upper and lower limits of the angular width of the incident beam (at ).
	Angular width () of the incident beam, set to be 0.34′′ in this paper.
	Middle position [] in the incident-beam angular width .
	Variation in the effective deviation angle owing to the vertical divergence of the beam, being the rate of the variation (= 0.028′′ mm^−1^).
*u*	Deviation parameter corresponding to the deviation angle , for the diffraction in the front crystal.
	Deviation parameters with respect to the diffraction of waves propagated in the transmitted- and diffracted-wave directions, respectively, after emerging from the front crystal.
	Observed intensities of the *O*- and *G*-wave diffraction moiré images, respectively.
,	Partial image intensities which are unrelated to moiré interference, but contribute to the total intensity (*O* image) .
,	Partial image intensities which are unrelated to moiré interference, but contribute to the total intensity (*G* image) .
,	Partial image intensities which are concerned in the formation of the *O*-wave moiré image.
,	Partial image intensities which are concerned in the formation of the *G*-wave moiré image.
,	Phases of moiré-fringe interference for the *O*- and *G*-wave moiré images, respectively.
	Gap phase difference (or gap phase).
	Moiré-fringe spacing.
	Elastic displacements in the *x* and the *y* directions, respectively, induced by stresses causing the LEC local strains.
,	Components of the LEC local strain in the front (*i* = 1) or rear (*i* = 2) crystals, related to the displacement .
	Component of the LEC local strain in the front (*i* = 1) or rear (*i* = 2) crystals, related to the displacement .

## References

[bb1] Hashimoto, H., Mannami, M. & Naiki, T. (1961). *Philos. Trans. R. Soc. A Math. Phys. Eng. Sci.* **253**, 490–516.

[bb2] Jäger, H. (1965). *Z. Angew. Phys.* **20**, 73–79.

[bb3] Jäger, H. (1966). *Z. Angew. Phys.* **21**, 543–548.

[bb4] Nagakura, S. (1972). Personal communication.

[bb5] Ohhashi, H. & Hirano, K. (2008). *Introduction to Synchrotron Beam Line Optical Techniques.* The Japanese Society for Synchrotron Radiation Research.

[bb6] Takeuchi, H. (1969). *Elasticity Theory.* Tokyo: Shokabo.

[bb7] Wilson, A. J. C. (1995). Editor. *International Tables for Crystallography*, Vol. C. Dordrecht: Kluwer.

[bb8] Yoshimura, J. (1984). *J. Appl. Cryst.* **17**, 426–434.

[bb9] Yoshimura, J. (1993). *Study of Nonprojectiveness of X-ray Moiré-fringed Images.* Report on research results to Grant-in-Aid for Scientific Research from The Ministry of Education and Science, Japan (No. 02452283).

[bb10] Yoshimura, J. (1996*a*). *Acta Cryst.* A**52**, 312–325.

[bb11] Yoshimura, J. (1996*b*). *J. Appl. Phys.* **80**, 2138–2141.

[bb12] Yoshimura, J. (1997*a*). *J. Appl. Phys.* **82**, 4697.

[bb13] Yoshimura, J. (1997*b*). *Acta Cryst.* A**53**, 810–812.

[bb14] Yoshimura, J. (1997*c*). *Acta Cryst.* A**53**, 813.

[bb15] Yoshimura, J. (2015). *Acta Cryst.* A**71**, 368–381. 10.1107/S2053273315004970 PMC4487425 25970298

[bb16] Yoshimura, J. (2019*a*). *Acta Cryst.* A**75**, 610–623. 10.1107/S2053273319004601 PMC6604514 31264645

[bb17] Yoshimura, J. (2019*b*). *Acta Cryst.* A**75**, 652–654. 10.1107/S2053273319006557 PMC6604513 31264649

[bb18] Yoshimura, J. & Hirano, K. (2009). *J. Synchrotron Rad.* **16**, 601–609. 10.1107/S0909049509029586 19713632

[bb19] Yoshimura, J. & Hirano, K. (2014). *Photon Factory Activity Report*, No. 31B, p. 516.

[bb20] Yoshimura, J., Hirano, K. & Zhang, X. (2001). *Photon Factory Activity Report*, No. 18, p. 187.

