# Supplementary material for: Theoretical study of the properties of X-ray diffraction moiré fringes. III. Theoretical simulation of previous experimental moiré images
Source: Acta Crystallogr A Found Adv. 2020 Jun 30;76(Pt 4):503–20. doi: 10.1107/S205327332000532X (PMC7330931; doi:10.1107/S205327332000532X)
Supplement: Supplementary file 1 [file a-76-00503-sup1.pdf]

FOUNDATIONS
ADVANCES

Volume 76 (2020)

Supporting information for article:

Theoretical study of the properties of X-ray diffraction moiré fringes. III. Theoretical simulation of previous experimental moiré images

Jun-ichi Yoshimura

Supporting information:

Derivation of the analytical expressions of strain distributions given in Appendix A

Calculation for the derivation of the analytical expressions in question was tried with reference to an exercise of elasticity theory (Takeuchi (1969); Exercise No. 64). We assume the following stresses applied on the boundary at $x = x_e$ of a semi-infinite elastic body in $x - x_e \geq 0$, as shown in Fig. 10 in the main text:

$$T_{yy} = P(y) = \begin{cases} A & (|y - y_o| \leq w) \\ 0 & (|y - y_o| > w) \end{cases} \quad \text{(S1}a\text{)} \qquad T_{xx} = Q(y) = \begin{cases} B & (|y - y_o| \leq w) \\ 0 & (|y - y_o| > w) \end{cases} \quad \text{(S1}b\text{)}$$

$$T_{xy} = T_{xz} = 0\,. \quad \text{(S1}c\text{)}$$

General solutions of the static displacements in the elastic body in the orthogonal coordinate system can be given as follows:

$$u_s = [-|\xi|\alpha + (1 - |\xi|x' - 2(\lambda_{\mathrm{L}} + 2\mu_{\mathrm{L}})\beta/(\lambda_{\mathrm{L}} + \mu_{\mathrm{L}}))]\exp(i\xi y - |\xi|x') \quad \text{(S2}a\text{)}$$

$$v_s = i\xi(\alpha + \beta x')\exp(i\xi y - |\xi|x')\,. \quad \text{(S2}b\text{)}$$

Here, u_s and v_s are the displacements in the x and y directions, respectively; α and β are undetermined constants, and ξ a variable; $x' = x - x_e$; λ_{L} and μ_{L} are Lamé's constants. From these u_s and v_s, the expressions of the stresses $T_{xx}\big|_{x'=0}$ and $T_{yy}\big|_{x'=0}$ are obtained through formulas of elasticity theory, as follows:

$$T_{xx}\big|_{x'=0} = 2\mu_{\mathrm{L}}|\xi|[|\xi|\alpha + (\lambda_{\mathrm{L}} + 2\mu_{\mathrm{L}})\beta/(\lambda_{\mathrm{L}} + \mu_{\mathrm{L}})]e^{i\xi y} = D(\xi)e^{i\xi y} \quad \text{(S3}a\text{)}$$

$$T_{yy}\big|_{x'=0} = -2\mu_{\mathrm{L}}|\xi|[|\xi|\alpha - \lambda_{\mathrm{L}}\beta/(\lambda_{\mathrm{L}} + \mu_{\mathrm{L}})]e^{i\xi y} = C(\xi)e^{i\xi y}\,. \quad \text{(S3}b\text{)}$$

From these equations, expressions of α and β are obtained, and they are substituted for α and β in equations (S2*a*) and (S2*b*). The expressions of u_s and v_s after the substitution are integrated with respect to ξ, and the existing u_s and v_s are redefined here as the resulting integrals, respectively:

$$u_s = -\int_{-\infty}^{+\infty}\left\{\frac{\mu_{\mathrm{L}}C(\xi) + (2\lambda_{\mathrm{L}} + 3\mu_{\mathrm{L}})D(\xi)}{4\mu_{\mathrm{L}}(\lambda_{\mathrm{L}} + \mu_{\mathrm{L}})|\xi|} + \frac{x'}{4\mu_{\mathrm{L}}}(C(\xi) + D(\xi))\right\}e^{i\xi y - |\xi|x'}d\xi \quad \text{(S4}a\text{)}$$

$$v_s = \int_{-\infty}^{+\infty}\left\{\frac{i\xi[-(\lambda_{\mathrm{L}} + 2\mu_{\mathrm{L}})C(\xi) + \lambda_{\mathrm{L}}D(\xi)]}{4\mu_{\mathrm{L}}(\lambda_{\mathrm{L}} + \mu_{\mathrm{L}})|\xi|^2} + \frac{i\xi x'}{4\mu_{\mathrm{L}}|\xi|}(C(\xi) + D(\xi))\right\}e^{i\xi y - |\xi|x'}d\xi\,. \quad \text{(S4}b\text{)}$$

The calculation is further continued by setting

$$C(\xi) = A\sin(w\xi)/\pi\xi\,, \quad \text{(S5}a\text{)} \qquad D(\xi) = B\sin(w\xi)/\pi\xi\,, \quad \text{(S5}b\text{)}$$

and we obtain the following results:

$$u_s = -\int_0^{+\infty}\left\{\frac{[\mu_{\mathrm{L}}A + (2\lambda_{\mathrm{L}} + 3\mu_{\mathrm{L}})B]}{2\pi\mu_{\mathrm{L}}(\lambda_{\mathrm{L}} + \mu_{\mathrm{L}})}\bullet\frac{e^{-x'\xi}}{\xi^2} + \frac{x'}{2\pi\mu_{\mathrm{L}}}(A + B)\bullet\frac{e^{-x'\xi}}{\xi}\right\}\cos y\xi\sin w\xi d\xi \quad \text{(S6}a\text{)}$$

$$v_s = \left\{ \frac{(\lambda_\mathrm{L} + 2\mu_\mathrm{L}) A - \lambda_\mathrm{L} B}{4\pi\mu_\mathrm{L} (\lambda_\mathrm{L} + \mu_\mathrm{L})} \right\} \left[(y+w) \tan^{-1}\left(\frac{y+w}{x'} \right) - (y-w) \tan^{-1}\left(\frac{y-w}{x'} \right) \right]$$

$$- \left\{ \frac{(2\lambda_\mathrm{L} + 3\mu_\mathrm{L}) A + \mu_\mathrm{L} B}{8\pi\mu_\mathrm{L} (\lambda_\mathrm{L} + \mu_\mathrm{L})} \right\} x' \log\left[\frac{x'^2 + (y+w)^2}{x'^2 + (y-w)^2} \right]. \qquad (\mathrm{S6}b)$$

From these expressions of displacements, we obtain the expressions for components of strain as given in Appendix A in the main text. The strains caused by image force on the top and bottom edges of the crystals were not taken into account, since the equations become lengthy even if the accuracy of calculation is improved to some extent.

Reference

Takeuchi, H. (1969). *Elasticity Theory*. Tokyo: Shokabo [In Japanese].
